# The clinical effectiveness of sertraline in primary care and the role of depression severity and duration (PANDA): a pragmatic, double-blind, placebo-controlled randomised trial

**DOI:** 10.1016/S2215-0366(19)30366-9

**Published:** 2019-11

**Authors:** Gemma Lewis, Larisa Duffy, Anthony Ades, Rebekah Amos, Ricardo Araya, Sally Brabyn, Katherine S Button, Rachel Churchill, Catherine Derrick, Christopher Dowrick, Simon Gilbody, Christopher Fawsitt, William Hollingworth, Vivien Jones, Tony Kendrick, David Kessler, Daphne Kounali, Naila Khan, Paul Lanham, Jodi Pervin, Tim J Peters, Derek Riozzie, George Salaminios, Laura Thomas, Nicky J Welton, Nicola Wiles, Rebecca Woodhouse, Glyn Lewis

**Affiliations:** a Division of Psychiatry, University College London, London, UK; b Bristol Medical School, University of Bristol, Bristol, UK; c Institute of Psychology Health and Society, University of Liverpool, Liverpool, UK; d Institute of Psychiatry, Psychology and Neuroscience, King's College London, London, UK; e Department of Health Sciences, University of York, York, UK; f Centre for Reviews and Dissemination, University of York, York, UK; g Department of Psychology, University of Bath, Bath, UK; h Primary Care and Population Sciences, Faculty of Medicine, University of Southampton, Aldermoor Health Centre, Southampton, UK; i School of Nursing and Health Studies, University of Dundee, Dundee, UK; j Department of Liberal Arts, Durham University, Durham, UK

## Abstract

**Background:**

Depression is usually managed in primary care, but most antidepressant trials are of patients from secondary care mental health services, with eligibility criteria based on diagnosis and severity of depressive symptoms. Antidepressants are now used in a much wider group of people than in previous regulatory trials. We investigated the clinical effectiveness of sertraline in patients in primary care with depressive symptoms ranging from mild to severe and tested the role of severity and duration in treatment response.

**Methods:**

The PANDA study was a pragmatic, multicentre, double-blind, placebo-controlled randomised trial of patients from 179 primary care surgeries in four UK cities (Bristol, Liverpool, London, and York). We included patients aged 18 to 74 years who had depressive symptoms of any severity or duration in the past 2 years, where there was clinical uncertainty about the benefit of an antidepressant. This strategy was designed to improve the generalisability of our sample to current use of antidepressants within primary care. Patients were randomly assigned (1:1) with a remote computer-generated code to sertraline or placebo, and were stratified by severity, duration, and site with random block length. Patients received one capsule (sertraline 50 mg or placebo orally) daily for one week then two capsules daily for up to 11 weeks, consistent with evidence on optimal dosages for efficacy and acceptability. The primary outcome was depressive symptoms 6 weeks after randomisation, measured by Patient Health Questionnaire, 9-item version (PHQ-9) scores. Secondary outcomes at 2, 6 and 12 weeks were depressive symptoms and remission (PHQ-9 and Beck Depression Inventory-II), generalised anxiety symptoms (Generalised Anxiety Disorder Assessment 7-item version), mental and physical health-related quality of life (12-item Short-Form Health Survey), and self-reported improvement. All analyses compared groups as randomised (intention-to-treat). The study is registered with EudraCT, 2013-003440-22 (protocol number 13/0413; version 6.1) and ISRCTN, ISRCTN84544741, and is closed to new participants.

**Findings:**

Between Jan 1, 2015, and Aug 31, 2017, we recruited and randomly assigned 655 patients—326 (50%) to sertraline and 329 (50%) to placebo. Two patients in the sertraline group did not complete a substantial proportion of the baseline assessment and were excluded, leaving 653 patients in total. Due to attrition, primary outcome analyses were of 550 patients (266 in the sertraline group and 284 in the placebo group; 85% follow-up that did not differ by treatment allocation). We found no evidence that sertraline led to a clinically meaningful reduction in depressive symptoms at 6 weeks. The mean 6-week PHQ-9 score was 7·98 (SD 5·63) in the sertraline group and 8·76 (5·86) in the placebo group (adjusted proportional difference 0·95, 95% CI 0·85–1·07; p=0·41). However, for secondary outcomes, we found evidence that sertraline led to reduced anxiety symptoms, better mental (but not physical) health-related quality of life, and self-reported improvements in mental health. We observed weak evidence that depressive symptoms were reduced by sertraline at 12 weeks. We recorded seven adverse events—four for sertraline and three for placebo, and adverse events did not differ by treatment allocation. Three adverse events were classified as serious—two in the sertraline group and one in the placebo group. One serious adverse event in the sertraline group was classified as possibly related to study medication.

**Interpretation:**

Sertraline is unlikely to reduce depressive symptoms within 6 weeks in primary care but we observed improvements in anxiety, quality of life, and self-rated mental health, which are likely to be clinically important. Our findings support the prescription of SSRI antidepressants in a wider group of participants than previously thought, including those with mild to moderate symptoms who do not meet diagnostic criteria for depression or generalised anxiety disorder.

**Funding:**

National Institute for Health Research.

## Introduction

Depression is a major contributor to the global burden of disease and by 2030 is predicted to be the leading cause of disability in high-income countries.^1^ People with depression are usually managed in primary care and SSRI antidepressants are often the first-line treatment.^2^, ^3^ Prescriptions for antidepressants have risen dramatically in high-income countries over the past decade (70·9 million items were prescribed in England in 2018), leading to concerns that they are overprescribed.^4^

Existing evidence on the clinical effectiveness of antidepressants has been comprehensively summarised in a recent network meta-analysis, which reported small benefits of all antidepressants compared with placebo.^5^ However, the constituent trials had many methodological limitations, were usually done decades ago for regulatory purposes, and generally were of poor quality, with 82% at moderate or high risk of bias.^5^ Larger, more recent placebo-controlled trials reported smaller antidepressant effect sizes, perhaps reflecting more rigorous methods.^5^

A more important criticism of the existing evidence is that most trials have used eligibility criteria based on diagnoses and severity. Primary care physicians see patients whose depressive symptoms range from mild to severe.^2^, ^6^ Milder depressive symptoms can still cause distress and impairment,^7^ and it is common for primary care physicians to prescribe antidepressants when diagnostic criteria are not met.^2^, ^8^ The use of antidepressants has increased and broadened since the original regulatory trials were done. Most antidepressants are now prescribed in primary care without any standardised assessment of depressive symptoms. It is difficult to generalise existing results to patients currently receiving treatment for depression.

Some studies have argued that antidepressants are more effective for patients with more severe symptoms^9^, ^10^ but most,^11^, ^12^, ^13^, ^14^ although not all,^15^ meta-analyses of individual patient data do not support this. Systematic reviews on the effectiveness of antidepressants for patients with less severe depression have led to contradictory findings.^16^, ^17^ The THREAD study was done in UK primary care and found small beneficial effects of SSRIs in patients with mild to moderate depressive symptoms, but comparison was with usual care rather than placebo.^18^

**Research in context**

**Evidence before this study**

Existing evidence on the effectiveness of antidepressants has been comprehensively summarised in a recent network meta-analysis by Cipriani and colleagues, which reported small benefits of all antidepressants compared with placebo. This meta-analysis included double-blind, randomised controlled trials published up to Jan 8, 2016, of adults meeting diagnostic criteria for depression. We updated this search for articles published up to Jan 31, 2019. We also searched for trials including patients not meeting diagnostic criteria for depression. We searched PubMed and the Cochrane Central Register of Controlled Trials, for studies published in English, with the search terms “depress*” OR “dysthymi*” OR “adjustment disorder” OR “mood disorder” OR “affective disorder” OR “depress* symptoms” AND “antidepress*”. We did not find any individual trials of antidepressants compared with placebo in people with depression published since the review by Cipriani and colleagues. We found two systematic reviews of patients with subthreshold depressive symptoms. Most trials were done in secondary care, with patients who met diagnostic criteria and specified severity thresholds. These trials had many methodological limitations, were usually done several decades ago for regulatory purposes, and were generally of poor quality, with 82% rated by Cipriani and colleagues as having a moderate or high risk of bias. Some primary care trials have been done, but with similar limitations. Most studies of patients with subthreshold depression were low quality and reported inconsistent findings.

**Added value of this study**

To our knowledge, the PANDA trial is the largest placebo-controlled trial of an antidepressant not funded by the pharmaceutical industry. We did not restrict eligibility by specifying lower or higher thresholds of depression severity but relied instead upon clinical uncertainty. This improved generalisability to the relevant population in primary care. In our primary analyses, we did not find convincing evidence that sertraline led to clinically important reductions in depressive symptoms within six weeks, although there was weak evidence of a small benefit at 12 weeks. In secondary analyses, we found evidence that sertraline led to reduced anxiety symptoms, better mental health-related quality of life, and self-reported improvements in mental health.

**Implications of all the available evidence**

Sertraline leads to reduced anxiety symptoms and self-reported improvements in mental health within 6 weeks, but any effect on depressive symptoms takes longer to emerge and is more modest. Our findings support the prescription of SSRI antidepressants in a wider group of participants than previously thought, including those with mild to moderate symptoms who do not meet diagnostic criteria for depression or generalised anxiety disorder. Clinicians and patients should be aware of the symptoms that are likely to improve so that they can consider alternative management of depressive symptoms that might not respond.

Duration of depression, as well as severity, might help determine whether antidepressants could be beneficial.^19^ Antidepressants can be effective for dysthymia—depressive symptoms not meeting diagnostic criteria but present for 2 years or more.^20^ Dysthymia has been criticised for conflating severity and duration.^19^ To our knowledge, no previous trial has investigated the association between depression duration and antidepressant response.

In this study, we report on the clinical effectiveness of sertraline in a large sample of patients who presented to primary care with depressive symptoms. We also explored the influence of depression severity and duration. We did not restrict eligibility by specifying lower or higher thresholds of depression severity but relied instead upon clinical uncertainty,^21^, ^22^ aiming to improve generalisability to the population receiving antidepressants in primary care.

## Methods

### Study design and participants

The PANDA study was a pragmatic, multicentre, double-blind, placebo-controlled randomised trial of patients from 179 primary care surgeries in four UK cities (Bristol, Liverpool, London, and York).^23^ Patients were eligible for inclusion if they were aged 18 to 74 years and there was uncertainty (from general practitioner [GP] and patient) about the possible benefit of an antidepressant. Use of clinical uncertainty as an entry criterion avoids diagnostic or severity criteria and leads to a sample that is more generalisable to the population currently receiving antidepressant medication. Clinical uncertainty relies upon variation between doctors in how they make decisions and, with a sufficient number of doctors, this variation in practice leads to a wide and heterogeneous group of participants in the trial, reflecting clinical equipoise within the field.^22^ In our case, because primary care physicians do not use standardised assessments of depression, we expected trial participants to have a range of depressive symptom severity. Clinical uncertainty is also the ethical basis for randomised controlled trials.^22^, ^24^, ^25^ Exclusion criteria were antidepressant treatment in past 8 weeks; comorbid psychosis, schizophrenia, mania, hypomania, bipolar disorder, dementia, eating disorder, or major alcohol or substance abuse; and medical contraindications for sertraline.^23^

There were two methods of recruitment to the study. First, patients were referred during GP consultation. Second, GP practices searched computerised records for patients who presented with depression or depressive symptoms in the past two years. Patients were told that they had been referred to the study because their GP had identified them as having depression or low mood. A researcher then contacted patients by telephone to confirm eligibility. Since it was possible that some patients might not currently have depressive symptoms, researchers asked “firstly, I need to ask you do you want treatment for your depression at the moment?”. If the answer was no, the patient was excluded. Before randomisation, the patient's GP was asked to approve inclusion in the trial and could exclude the patient if there was not clinical uncertainty.

All participants provided written informed consent. Ethics approval was from the National Research Ethics Service committee, East of England—Cambridge South (ref 13/EE/0418). The results presented here focus on the treatment effect of sertraline in primary care and are part of a larger research programme (NIHR RP-PG-0610-10048).

### Randomisation and masking

Participants were randomly assigned (1:1) to sertraline or placebo by PRIMENT Clinical Trials Unit (CTU) with a remote computer-generated code, and were stratified by severity, duration, and site with random block length. Prespecified thresholds for stratification were total score (0–11, 12–19, and ≥20) and depression duration (<2 years or ≥2 years) at baseline on the Clinical Interview Schedule—Revised (CIS-R).^26^ Sertraline 50 mg tablets and placebo were encapsulated and were identical. Researchers and statisticians were masked to treatment allocation until completion of analyses. Primary care physicians were told the treatment allocation when participants finished their final assessment or withdrew from study medication, so they could discuss further treatment.

### Procedures

Patients received one capsule (sertraline 50 mg or placebo orally) daily for one week then two capsules daily for up to 11 weeks, consistent with evidence on optimal dosages for efficacy and acceptability.^27^ After six weeks, medication could be increased to three capsules in consultation with the local principal investigator. Trial medication was sent by a trial pharmacy to the patient's primary care physician (or home) after randomisation. Baseline assessments before randomisation and follow-up assessments at 2 weeks, 6 weeks, and 12 weeks after randomisation took place in patients' homes, primary care surgeries, or universities.^23^

We assessed patients' depressive symptoms with the Patient Health Questionnaire, 9-item version (PHQ-9; available range 0–27, higher scores indicating more severe symptoms), a self-report measure of depressive symptoms in the past 2 weeks that is widely used in primary care.^28^ Self-report questionnaires avoid the possibility of observer bias associated with clinically rated outcomes^29^, ^30^ and the PHQ-9 might be more responsive to change than other self-report measures.^31^, ^32^ We also assessed depressive symptoms with the Beck Depression Inventory (BDI-II). The BDI-II is a self report 21-item scale that assesses the severity of depressive symptoms in the past 2 weeks. Scores range from 0–63, with higher scores indicating more severe symptoms. We assessed generalised anxiety symptoms with the Generalised Anxiety Disorder Assessment 7-item version (GAD-7). The GAD-7 is a self-report measure of the severity of anxiety symptoms in the past 2 weeks, with possible scores ranging from 0–21 and higher scores indicating more severe symptoms. We derived separate physical and mental health-related quality of life scores with the 12-item Short-Form Health Survey (SF-12). Possible scores range from 0 to 100, with higher scores indicating better quality of life.

We used a five-item self-report adherence scale developed for the CoBalt study and a binary variable indicating at least 80% adherence.^33^ Patients were also asked about serious adverse events at each follow-up visit by use of open-ended questions. Physical symptoms that could be SSRI side-effects were recorded with a modified Toronto scale.^34^

The CIS-R is a computerised, self-administered, fully structured interview measuring 14 common mental disorder symptom groups.^26^ The CIS-R generates diagnoses meeting ICD-10 criteria for depressive or anxiety episodes, a total common mental disorders score, and a depression severity score (available range 0–21) created by the sum of the following five symptoms: depression, depressive ideas, fatigue, concentration, and sleep problems. The total score in three categories was used for stratification. The severity score was used for the hypothesis that the treatment effect varied according to the severity of depressive symptoms at baseline. The CIS-R asks about duration of depressive symptoms with the following categories: less than 2 weeks, between 2 weeks and 6 months, between 6 months and 1 year, between 1 year and 2 years; between 2 years and 5 years; between 5 years and 10 years, and more than 10 years. These categories were dichotomised into less than 2 years and 2 years or more.

### Outcomes

The primary outcome was total score at 6 weeks on the PHQ-9.

Secondary outcomes were analysed as repeated measures across 2-week, 6-week, and 12-week follow-up assessments and included PHQ-9 and BDI-II total scores and remission (defined as a score of <10 on each measure), GAD-7 total scores, mental and physical health-related quality-of-life scores with the SF-12, and self-reported improvement in mental health. Self-reported improvement was assessed by asking patients, “compared to when we last saw you, how have your moods and feelings changed?”. Responses were “I feel a lot better”, “I feel slightly better”, “I feel about the same”, “I feel slightly worse”, or “I feel a lot worse”.^35^, ^36^ We created a binary variable of feeling the same or worse (0) and feeling better (1). Patients also completed the five-level EQ-5D instrument (for economic analyses) and emotional processing tasks, the results of which will be reported separately.

### Statistical analyses

When originally devised, the primary aim of the PANDA trial was to investigate the severity and duration of depressive symptoms associated with a clinically important response to sertraline, as stated in the protocol paper^23^ and on ISCRTN. However, towards the later stages of designing the trial and when formulating the detailed analysis plan (uploaded before any analyses were done to UCL Discovery and approved by the trial steering committee), it became apparent that we would have insufficient statistical power to estimate plausible interaction effects that would allow us to investigate those aims. Therefore, our power calculation^23^ and primary analysis (as stated in the analysis plan) are based on a primary aim to examine the clinical effectiveness of sertraline versus placebo. Interactions between severity and duration at baseline and treatment allocation were planned as exploratory. Our protocol paper has described the power calculation in detail.^23^ Assuming a two-sided α of 5%, power of 90%, and 10% attrition, 547 participants were required to detect an 11% (relative) difference in scores between sertraline and placebo.

Analyses and reporting were in line with CONSORT guidelines. Primary and secondary outcomes were described in the approved protocol before the study started (appendix pp 59). The measure of self-reported improvement was added as a secondary outcome after the study started but before any analyses were done (appendix pp 25). When the study was registered on ISCRTN, the primary outcome was listed. Secondary outcomes were added later and were described and approved in the study protocol. All analyses compared groups as randomised (intention-to-treat). We compared the proportion of patients in each group who were categorised as adherent with Pearson's χ^2^ test. Physical symptoms that could be SSRI side-effects were compared by group with independent *t*-tests. Analyses of adherence and physical symptoms were done at each follow-up visit. We report serious adverse events during the trial according to treatment allocation.

The primary analysis was a linear regression of log-transformed PHQ-9 scores at 6 weeks, adjusted for baseline PHQ-9 score and stratification variables (severity assessed by CIS-R in three categories, duration in two categories, and site). Regression coefficients from this model were the difference in geometric means of log-transformed PHQ-9 scores between randomised groups. We exponentiated this regression coefficient to report the treatment effect as a ratio of the (geometric) mean PHQ-9 score at 6 weeks in those allocated to sertraline to the (geometric) mean PHQ-9 score at 6 weeks in those allocated to placebo. The null value was 1 and proportions below 1 indicated fewer symptoms in the sertraline group. For example, a coefficient of 0·89 reflects PHQ-9 scores that were 11% lower in the sertraline than placebo group.

The proportional scale was chosen on the basis of model comparison between different scales. This choice was supported by previous work showing benefits for choosing the scale. These benefits include variance stabilisation and overcoming the issue of baseline dependence.^35^, ^37^ A proportional model is also plausible, as it seems unlikely that a treatment would lead to the same difference in means in those starting at a high score compared with those with a low score.

Given our interest in the relationship between severity and treatment effect, we included baseline depression CIS-R scores in the primary analysis. The primary analysis was also done without the CIS-R depression score, to examine the possible influence of collinearity. We investigated how robust findings were to the model specification by comparison with a Poisson model.

We calculated linear multilevel models for continuous secondary outcomes (PHQ-9, BDI-II, GAD-7, and SF-12 physical and mental health quality of life) with log-transformed PHQ-9, BDI-II, and GAD-7 scores and raw SF-12 scores, as stated in the analysis plan. Logistic multilevel models were calculated for binary secondary outcomes (remission on PHQ-9 and BDI-II, and self-reported improvement). Models included continuous baseline measurement of the outcome, CIS-R depression severity score, and stratification variables. For primary and secondary outcomes, we report interactions between treatment allocation and baseline CIS-R depression severity score and duration.

For sensitivity analyses, we reported primary and secondary outcomes after adjustment for variables that were possibly imbalanced at baseline, which were identified using descriptive statistics. We investigated possible clustering within surgeries by calculating the intraclass correlation coefficient using multilevel models, with a random intercept for surgeries. We reported the treatment effect from this multilevel model to assess whether clustering had an influence.

We did two sensitivity analyses to investigate the possible effect of missing data. First, we adjusted for baseline variables associated with missing outcome data. Second, we used multiple imputation by chained equations to replace missing data. We assumed missingness was dependent on observed data and imputed 50 datasets. Imputation models included variables from our models and variables associated with missing data, with pooled results obtained using Rubin's rules.^38^ Our post-hoc analyses are detailed in the appendix (p 4).

The study was overseen by a data monitoring committee. All analyses were done in STATA version 15. The study is registered with EudraCT, 2013-003440-22 (protocol number 13/0413; version 6.1), and ISRCTN, ISRCTN84544741.

### Role of the funding source

The funder of the study had no role in study design, data collection, data analysis, data interpretation, or writing of the report. The corresponding author had full access to all the data in the study and had final responsibility for the decision to submit for publication.

## Results

Between Jan 1, 2015, and Aug 31, 2017, we recruited and randomly assigned 655 patients—326 (50%) to sertraline and 329 (50%) to placebo (figure). Two patients in the sertraline group did not complete a substantial proportion of the baseline assessment and were excluded, leaving 653 patients in total included in analyses. Patients were recruited from 179 GP surgeries (appendix p 7). Patients who were invited to participate and those who participated were similar in age and sex (appendix p 8).

**Figure:**
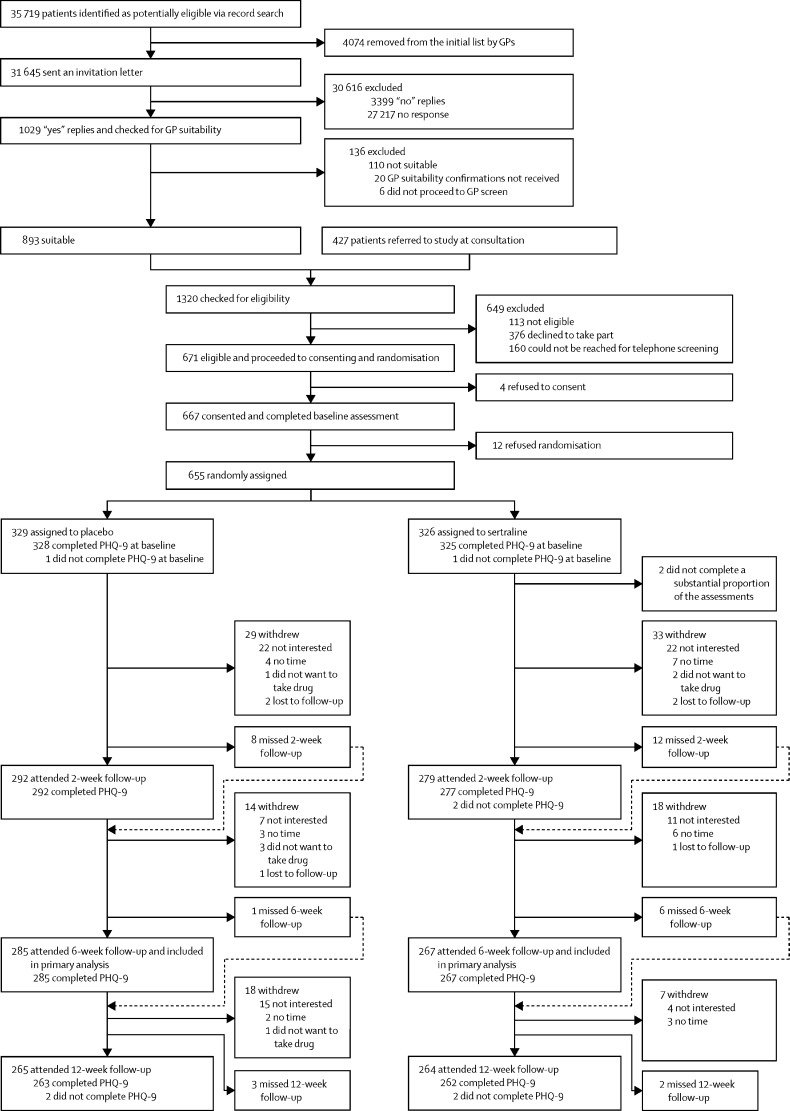
Trial profile GP=general practitioner. PHQ-9=Patient Health Questionnaire, 9-item version.

The proportions of patients attending each follow-up visit were similar in both trial groups and we found no statistical evidence for a difference in attrition by group (figure). Missing follow-up data were more likely in patients who were recruited in London, younger, ethnic minority, single, having financial difficulties, and those with no formal qualifications, and higher baseline PHQ-9, BDI-II, GAD-7, and life-event scores (appendix pp 9–10).

Baseline characteristics of the sample were similar between treatment groups (table 1). Overall, the mean patient age was 39·7 years (SD 14·96), 384 (59%) participants were female, and 433 (66%) were employed. On the CIS-R, 355 (54%) patients met ICD-10 criteria for depression, 299 (46%) patients met generalised anxiety criteria, 197 (30%) met both depression and generalised anxiety disorder criteria, and 96 (15%) met criteria for mixed anxiety and depressive disorder (scoring 12 or more but not meeting diagnostic criteria for depression or anxiety).^39^ 99 (15%) patients did not meet any diagnostic criteria for depression or anxiety. The mean PHQ-9 score was 12·0 (SD 5·8) and 405 (62%) patients exceeded a score of 10 (the recommended threshold for clinical use). 391 (60%) patients had received antidepressants previously and 522 (80%) patients reported previous depression. In the sertraline group, there was a higher proportion of women, and lower proportions of those who were married or living as married and meeting ICD-10 depression criteria.

**Table 1:** Baseline characteristics

		**Sertraline (n=324)**	**Placebo (n=329)**	**Overall (n=653)**
Site*				
	Bristol	131 (40%)	134 (41%)	265 (41%)
	Liverpool	58 (18%)	58 (18%)	116 (18%)
	London	69 (21%)	73 (22%)	142 (22%)
	York	66 (20%)	64 (19%)	130 (20%)
CIS-R total score*				
	0–11	67 (21%)	62 (19%)	129 (20%)
	12–19	84 (26%)	89 (27%)	173 (26%)
	≥20–49	173 (53%)	178 (54%)	351 (54%)
CIS-R depression duration (years)*				
	<2	218 (67%)	221 (67%)	439 (67%)
	≥2	106 (33%)	108 (33%)	214 (33%)
Age (years)				
	18–34	132 (41%)	134 (41%)	266 (41%)
	35–54	125 (39%)	134 (41%)	259 (40%)
	55–74	67 (21%)	61 (19%)	128 (20%)
Sex				
	Female	203 (63%)	181 (55%)	384 (59%)
	Male	121 (37%)	148 (45%)	269 (41%)
ICD-10 CIS-R depression diagnosis†				
	Yes	167 (52%)	188 (57%)	355 (54%)
	No	156 (48%)	141 (43%)	297 (46%)
ICD-10 CIS-R anxiety diagnosis†				
	Yes	144 (45%)	155 (47%)	299 (46%)
	No	179 (55%)	174 (53%)	353 (54%)
Ethnicity†				
	White	294 (91%)	285 (87%)	579 (89%)
	Ethnic minority	29 (9%)	44 (13%)	73 (11%)
Marital status†				
	Married or living as married	116 (36%)	139 (42%)	255 (39%)
	Single	152 (47%)	144 (44%)	296 (45%)
	Separated, divorced, or widowed	55 (17%)	46 (14%)	101 (15%)
Employment status†				
	In paid employment	209 (65%)	224 (68%)	433 (66%)
	Not employed	114 (35%)	105 (32%)	219 (34%)
Financial difficulty†				
	Living comfortably or doing alright	180 (56%)	184 (56%)	364 (56%)
	Just about getting by	101 (31%)	103 (31%)	204 (31%)
	Finding it difficult or very difficult	42 (13%)	42 (13%)	84 (13%)
Highest educational qualification†				
	A Level or higher	216 (67%)	234 (71%)	450 (69%)
	GCSE, standard grade, or other	92 (28%)	77 (23%)	169 (26%)
	No formal qualification	15 (5%)	18 (5%)	33 (5%)
Depression in the past†				
	Yes	259 (80%)	263 (80%)	522 (80%)
	No	64 (20%)	66 (20%)	130 (20%)
Antidepressant in the past†				
	Yes	191 (59%)	200 (61%)	391 (60%)
	No	132 (41%)	129 (39%)	261 (40%)
Patient Health Questionnaire, 9-item version total score (possible range 0–27)		11·79 (5·90)	12·20 (5·71)	12·00 (5·80)
CIS-R total score (possible range 0–64)		20·89 (10·09)	21·60 (10·19)	21·25 (10·14)
CIS-R depression severity score (possible range 0–21)‡		10·40 (4·95)	10·76 (4·84)	10·58 (4·90)
Beck Depression Inventory, second edition total score (possible range 0–63)		24·01 (10·54)	23·87 (10·07)	23·94 (10·30)
Generalised Anxiety Disorder Assessment, 7-item version score (possible range 0–21)		9·44 (5·39)	9·42 (5·17)	9·43 (5·28)
Social support score (possible range 1–24)		12·46 (4·01)	12·83 (3·63)	12·64 (3·82)
SF-12 mental health subscale (possible range 0–100)		32·96 (10·99)	31·98 (11·08)	32·47 (11·04)
SF-12 physical health subscale (possible range 0–100)		52·19 (9·36)	51·95 (10·04)	52·07 (9·70)
Number of life events in past 6 months		1·20 (1·20)	1·24 (1·19)	1·22 (1·19)
Number of physical symptoms in past 2 weeks		10·01 (4·44)	10·12 (5·42)	10·07 (5·42)
Frequency of physical symptoms (possible range 55–112)§		43·86 (11·13)	43·95 (10·80)	43·91 (10·96)

Data are n (%) or mean (SD). One person in the placebo group was missing the PHQ-9 score at baseline and one person in the sertraline group was missing the CIS-R depression score at baseline. CIS-R=Clinical Interview Schedule—Revised. SF-12= Short-Form Health Survey.

* The total CIS-R score assesses the severity of symptoms of common mental disorders. The total CIS-R score in three categories was a stratification variable at randomisation: 0–11 (minimal symptoms), 12–19 (moderate to severe symptoms), and ≥20 (severe symptoms).

† A CIS-R diagnosis used the criteria and threshold required to meet an ICD-10 clinical diagnosis of depression or anxiety. CIS-R data were missing for one person.

‡ The CIS-R depression severity score (possible range 0–21) assesses the severity of depressive symptoms.

§ How often during the past 2 weeks the patient had each symptom: 1 (not at all), 2 (several days), 3 (more than half the days), and 4 (nearly every day).

563 (86%) of 653 randomised patients provided the date they started medication (median 8 days after randomisation, IQR 6–11, range 1–70). There were delays with follow-up assessments so that these were carried out a median of 1 week, 5 weeks, and 11 weeks after patients started medication.

Adherence was greater than 80% at all timepoints and we observed no evidence of a difference in adherence between treatment groups (appendix p 11). More patients in the sertraline group than the placebo group thought they were taking sertraline at 2 weeks, 6 weeks, and 12 weeks (appendix p 11).

Due to attrition and missing data, 550 patients were included in the analysis of the primary outcome. Mean PHQ-9 scores at 6 weeks were 7·98 (SD 5·63) in patients allocated to sertraline and 8·76 (5·86) in patients allocated to placebo (table 2). After adjustment for baseline scores and stratification variables, the adjusted proportional difference between sertraline and placebo was 0·95 (95% CI 0·85–1·07; p=0·41; model 1; table 2). This result changed very little after adjustment for variables showing imbalance (model 2; table 2). We found little evidence of clustering by surgery (intracluster correlation coefficient 0·04, 95% CI 0·00–0·01) and no evidence that clustering altered the treatment effect (model 3; table 2). The treatment effect was similar after considering missing data (models 4, 5, 6; table 2).

**Table 2:** Analyses of primary outcome (log-transformed PHQ-9 scores at 6 weeks)

	**Sertraline**		**Placebo**		Adjusted proportional difference*(95% CI)	**p value**
	n	Mean (SD)	n	Mean (SD)		
Model 1†	266	7·98 (5·63)	284	8·76 (5·86)	0·95 (0·85–1·07)	0·41
Model 2‡	266	7·98 (5·63)	284	8·76 (5·86)	0·94 (0·84–1·05)	0·30
Model 3§	266	7·98 (5·63)	284	8·76 (5·86)	0·95 (0·85–1·05)	0·31
Model 4¶	266	7·98 (5·63)	282	8·81 (5·85)	0·94 (0·85–1·05)	0·28
Model 5‖	324	8·25 (5·81)	329	8·88 (5·87)	0·96 (0·86–1·08)	0·50
Model 6**	324	8·25 (5·81)	329	8·88 (5·87)	0·95 (0·85–1·07)	0·39

Means (SDs) are for non-log-transformed PHQ-9 scores at 6 weeks. One person in the placebo group was missing the PHQ-9 score at baseline and one person in the sertraline group was missing the CIS-R depression score at baseline. These individuals were excluded from the regression model of the primary outcome at 6 weeks. PHQ-9=Patient Health Questionnaire, 9-item version. CIS-R=Clinical Interview Schedule—Revised.

* All models use a log-transformed PHQ-9 score as the outcome. Adjusted proportional difference can be interpreted as the difference in scores between randomised groups expressed as a proportion (or percentage).

† Primary analysis model: intention-to-treat analysis adjusted for baseline PHQ-9 and CIS-R depression scores and stratification variables (total CIS-R score in three categories, duration of depressive episode in two categories, and site; n=550).

‡ Model 1 adjusted for variables showing imbalance at baseline (sex, ICD-10 depression diagnosis, marital status; n=550).

§ Model 1 adjusted for possible clustering effects using a random effect for general practices (n=550).

¶ Model 1 adjusted for baseline variables associated with missing outcome data (age, ethnicity, marital status, financial difficulty, ICD-10 anxiety diagnosis, used antidepressants in the past, Beck Depression Inventory, second edition score, number of life events, Generalised Anxiety Disorder Assessment, 7-item version score; n=550).

‖ Model 1 with missing data replaced by multiple imputation (n=653).

** The imputed model (model 5) adjusted for variables showing imbalance at baseline (sex, ICD-10 depression diagnosis, marital status; n=653).

We observed an association between baseline CIS-R depression severity and PHQ-9 scores at 6 weeks, with a 3% increase in PHQ-9 scores for each unit increase in CIS-R depression score (adjusted proportional difference 1·03, 95% CI 1·01–1·05; p=0·0046). We found no evidence of an association between baseline depression duration and PHQ-9 score at 6 weeks (1·08, 95% CI 0·96–1·22; p=0·19). We observed no evidence that treatment response varied with depression severity (p=0·9907) or duration (p=0·8203).

The result of the primary analysis did not change after we excluded baseline CIS-R depression scores to explore possible multicollinearity (adjusted proportional difference 0·96, 95% CI 0·85–1·07; p=0·41). The result of the primary analysis was consistent with a negative binomial model (chosen as an extension of the Poisson model because PHQ-9 variance at 6 weeks was larger than the mean).

The adjusted proportional difference in PHQ-9 scores across all timepoints was 0·93 (95% CI 0·86–1·01, p=0·11; table 3). At 12 weeks, PHQ-9 scores were 13% lower (0·87, 95% CI 0·79–0·97) in the sertraline group. We observed similar results for the BDI-II (table 3). We observed evidence that differences in GAD-7 scores became larger over time (p=0·0075). At 6 weeks, GAD-7 scores were 21% lower (adjusted proportional difference 0·79, 95% CI 0·70–0·89) in those allocated to sertraline than in those allocated to placebo. Mental health-related quality of life scores were higher (2·41, 95% CI 1·14–3·96, p=0·00021) in the sertraline group than in the placebo group, with no evidence that differences varied over time (p=0·22). We observed no evidence of a difference in physical health-related quality of life (table 3).

**Table 3:** Repeated measures analyses of continuous secondary outcomes at 2 weeks, 6 weeks, and 12 weeks

		**Sertraline**		**Placebo**		Adjusted proportional difference*(95% CI)	**p value**
		n	Mean (SD)	n	Mean (SD)		
**PHQ-9**							
Follow-up assessment (weeks)							
	2	277	9·94 (5·83)	292	10·32 (5·55)	0·96 (0·87 to 1·07)	..
	6	266	7·98 (5·63)	284	8·76 (5·86)	0·96 (0·86 to 1·07)	..
	12	262	6·90 (5·83)	263	8·02 (6·12)	0·87 (0·79 to 0·97)	..
Over time†		..	..	..	..	0·93 (0·86 to 1·01)	0·11
Group by time interaction		..	..	..	..	..	0·0905
**Beck Depression Inventory, second edition**							
Follow-up assessment (weeks)							
	2	273	18·77 (11·08)	286	19·10 (11·17)	0·99 (0·97 to 1·10)	..
	6	266	14·82 (10·44)	285	15·91 (10·74)	0·95 (0·85 to 1·07)	..
	12	256	12·44 (10·96)	259	14·78 (11·70)	0·84 (0·74 to 0·95)	..
Over time†		..	..	..	..	0·93 (0·84 to 1·21)	0·012
Group by time interaction		..	..	..	..	..	0·015
**GAD-7**							
Follow-up assessment (weeks)							
	2	277	7·55 (5·49)	291	8·16 (5·26)	0·91 (0·82 to 1·03)	..
	6	264	5·55 (5·19)	284	6·96 (5·24)	0·79 (0·70 to 0·89)	..
	12	263	4·95 (5·30)	263	6·27 (5·28)	0·77 (0·68 to 0·87)	..
Over time†		..	..	..	..	0·83 (0·75 to 0·91)	<0·0001
Group by time interaction		..	..	..	..	..	0·0075
**SF-12 mental health**							
Follow-up assessment (weeks)							
	2	275	37·32 (11·47)	291	35·37 (11·36)	1·58 (−0·10 to 3·26)	..
	6	254	41·95 (12·35)	277	38·67 (11·91)	2·90 (1·17 to 4·63)	..
	12	263	42·70 (12·91)	264	39·71 (11·87)	2·85 (1·12 to 5·47)	..
Over time†		..	..	..	..	2·41 (1·14 to 3·69)	0·0002
Group by time interaction		..	..	..	..	..	0·22
**SF-12 physical health**							
Follow-up assessment (weeks)							
	2	275	51·92 (9·18)	291	52·40 (6·64)	−0·71 (−1·75 to 0·34)	..
	6	245	51·98 (8·39)	277	51·76 (9·90)	−0·36 (−1·44 to 0·71)	..
	12	263	51·92 (8·53)	264	52·50 (9·99)	−0·89 (−1·96 to 0·19)	..
Over time†		..	..	..	..	−0·66 (−1·48 to 0·17)	0·12
Group by time interaction		..	..	..	..	..	0·79

PHQ-9=Patient Health Questionnaire, 9-item version. SF-12= Short-Form Health Survey. CIS-R=Clinical Interview Schedule—Revised.

* These models used a log-transformed PHQ-9 score as the outcome. Adjusted proportional differences can be interpreted as the difference in scores between randomised groups expressed as a proportion (or percentage). All multilevel models were adjusted for baseline measure of each outcome (continuous), baseline CIS-R depression severity score, and stratification variables (baseline total CIS-R score in three categories, duration of depressive episode in two categories, and site).

† Calculated using data from all three follow-ups combined.

We found evidence that sertraline led to increased odds of remission at 12 weeks (but not 6 weeks) and this evidence was stronger for BDI-II than PHQ-9 (table 4; appendix p 24). Patients in the sertraline group had increased odds of reporting feeling better than the same or worse (adjusted OR 1·96, 95% CI 1·45–2·63; p<0·0001), with no evidence that this difference varied over time (p=0·16).

**Table 4:** Repeated measures analyses of binary secondary outcomes at 2 weeks, 6 weeks, and 12 weeks

		**Sertraline**		**Placebo**		Adjusted*OR (95% CI)	**p value**
		n	n (%)	n	n (%)		
**Patient Health Questionnaire, 9-item version remission**							
Follow-up assessment (weeks)							
	2	277	145 (52%)	292	136 (47%)	1·36 (0·76–2·41)	..
	6	266	169 (63%)	284	164 (58%)	1·30 (0·72–2·34)	..
	12	262	190 (73%)	263	170 (65%)	1·75 (0·94–3·27)	..
Over time†		..	..	..	..	1·44 (0·93–2·22)	0·10
Group by time interaction		..	..	..	..	..	0·49
**Beck Depression Inventory, second edition remission**							
Follow-up assessment (weeks)							
	2	273	58 (21%)	286	58 (20%)	1·01 (0·52–1·98)	..
	6	266	94 (35%)	285	91 (32%)	1·28 (0·70–2·35)	..
	12	256	131 (51%)	259	102 (39%)	2·69 (1·46–4·97)	..
Over time†		..	..	..	..	1·58 (1·00–2·47)	0·049
Group by time interaction		..	..	..	..	..	0·012
**Feeling better (self-rated improvement)**							
Follow-up assessment (weeks)							
	2	279	110 (39%)	292	89 (30%)	1·64 (1·06–2·53)	..
	6	267	157 (59%)	285	132 (46%)	1·90 (1·24–2·91)	..
	12	264	156 (59%)	265	112 (42%)	2·42 (1·56–3·75)	..
Over time†		..	..	..	..	1·96 (1·45–2·63)	<0·0001
Group by time interaction		..	..	..	..	..	0·16

CIS-R=Clinical Interview Schedule—Revised.

* All multilevel models were adjusted for baseline measure of each outcome (continuous), baseline CIS-R depression severity score, and stratification variables (baseline total CIS-R score in three categories, duration of depressive episode in two categories, and site).

† Calculated using data from all three follow-ups combined.

Analysis of secondary outcomes was unaffected by adjustment for variables that were imbalanced at baseline (appendix pp 13–14). We observed no evidence that treatment response for secondary outcomes varied according to severity or duration of symptoms. Results of our post-hoc analyses are presented in the appendix (pp 15–24).

We recorded seven adverse events—four in the sertraline group and three in the placebo group (appendix p 12). Three adverse events were classified as serious—two in the sertraline group and one in the placebo group. One serious adverse event in the sertraline group was classified as possibly related to study medication and two in the placebo group were classified as not related or unlikely to be related (appendix p 12). We found no evidence of a difference in the number of physical symptoms between groups (appendix p 12). 59 patients chose to increase their dose to 150 mg—37 (11%) in the placebo group and 22 (7%) in the sertraline group (p=0·047). We are aware of three incidents in which the capsule was broken. One of these incidents was intentional, although the participant drew the wrong conclusion about allocation. The other two incidents were inadvertent. The three affected participants were withdrawn from study medication once we were aware of these incidents.

## Discussion

In our primary analysis, we did not find convincing evidence that sertraline led to a greater reduction in depressive symptoms compared with placebo within 6 weeks. However, in our secondary analyses we found weak evidence that sertraline reduced depressive symptoms at 12 weeks compared with placebo. This effect was small, but does not exclude the possibility of a clinically important effect on depressive symptoms by 12 weeks.

In our secondary analyses, we found evidence that sertraline reduced generalised anxiety symptoms at 6 weeks and 12 weeks compared with placebo. We also found evidence that sertraline led to better mental (but not physical) health-related quality of life and self-rated improvement in mental health.

Although results from secondary analyses should be interpreted with caution, statistical evidence for all our secondary outcomes (except depression) was strong. In particular, self-reported improvements in mental health can be used to calculate minimal clinically important differences and can be regarded as patient-centred indicators of clinically meaningful change.^40^ Depressive and anxiety symptoms often occur together and comorbid anxiety symptoms have been associated with poorer quality of life in people with depressive symptoms.^41^, ^42^ We have found important evidence that sertraline can lead to clinical benefits for patients in primary care with depressive symptoms. However, these benefits occur mainly through improvements in anxiety symptoms and quality of life rather than reductions in depressive symptoms.

We found no evidence that severity or duration of depressive (or anxiety) symptoms affected treatment response, although analyses using interaction terms lacked statistical power. We found that duration of symptoms was not associated with outcome once severity of symptoms had been accounted for, suggesting that an interaction with treatment response is unlikely.

Sertraline has a similar pharmacological profile to other SSRIs and acts via a similar mechanism.^5^ Therefore, we would expect our results to apply to other SSRIs when used in this population. Generalising our findings to antidepressants of other classes is more difficult, although there are similarities in the mechanism of action of all commonly used antidepressants.^43^

To our knowledge, the PANDA trial is the largest individual placebo-controlled trial of an antidepressant not funded by the pharmaceutical industry. Our trial had more attrition than predicted but recruited more participants than our target and had power to detect an 11% reduction in symptoms—enough to detect a clinically important difference. Clinical trials are often criticised for using narrow inclusion criteria, which can reduce external validity.^25^ We used clinical uncertainty as an entry criterion, which avoids reliance upon diagnostic or severity criteria that have never been validated as an indication for antidepressants. Participants in our study ranged from those with very few depressive symptoms to those with severe depressive symptoms, therefore our results are more readily generalisable to the population currently receiving antidepressants in primary care than are the results of previous trials. Many people in our sample had very severe depression, suggesting that there is uncertainty about prescription of antidepressants in primary care at all levels of severity.

Use of clinical uncertainty as an entry criterion could have potential disadvantages. If patients with few depressive symptoms were less likely to respond to antidepressants, this might have reduced the treatment effect, but we did not find any suggestion of this. We also observed strong evidence of a treatment effect for anxiety symptoms despite our broad inclusion criteria.

Reliance upon clinical uncertainty will exclude those for whom a clinician is certain that antidepressants are indicated. We would expect people with the most severe depression to be somewhat underrepresented in the trial. However, this effect should be modest given the very wide range of depression severity in our sample. Use of clinical uncertainty as an entry criterion requires many clinicians to participate in a study to encompass the whole range of clinical practice. We included 179 practices from four UK cities, therefore it is unlikely that we are reflecting idiosyncratic practice. Instead, our sample most likely reflects the diverse decisions made by many doctors over a broad range of uncertainty.

There are marked similarities between the behaviour of doctors and patients in primary care within high-income countries, despite divergent health systems. Antidepressant prescribing has increased in all high-income countries over the past two decades.^44^ A similar trial in other high-income countries would probably identify participants over a wide range of depression severities and include many people with anxiety symptoms. Our main conclusions about the benefits of sertraline on anxiety rather than depressive symptoms should be generalisable to other high-income countries.

In the PANDA trial, 115 (46%) participants on sertraline thought they were taking the active drug at 6 weeks compared with 52 (19%) participants on placebo. In other SSRI trials that have assessed patient beliefs about the medication that they are taking,^45^, ^46^, ^47^, ^48^ only one study^45^ found evidence that patients can distinguish active treatment from placebo. We considered two possible explanations for this finding in our trial. First, patients detected an improvement in their symptoms, therefore thought they were taking sertraline. Second, patients could detect being on sertraline because of physical or psychological effects that we did not measure. We found no evidence that side-effects differed by study group, therefore it is unlikely that reported adverse effects systematically led to a difference in patients guessing whether they were on sertraline or placebo.

The possibility that adverse effects of antidepressants could lead to unblinding and an inadvertent placebo effect has been raised previously.^49^ Such a placebo effect should lead to a difference in all outcomes. In our trial, anxiety, quality of life, and self-reported improvement measures showed greater changes than depressive symptom measures. This observation reduces the likelihood that differences between sertraline and placebo were explained purely by the possibility of unblinding.

Medication was encapsulated so participants could, in principle, break open the capsule and discover if there was a tablet included. We are aware of three incidents when this occurred. All three participants stopped the study medication once this had occurred and we do not think the encapsulation of the medication had an overall effect on the blinding of assessments in the trial.

Patients from record searches had less severe depressive and anxiety symptoms but were more likely to have taken antidepressants previously. These differences had no influence on treatment effects (appendix pp 19–21). Current practice in UK primary care is to engage with patients' views and to share decision making.^50^ Therefore, patients who present to clinicians with mental health problems and ask for antidepressants will usually be prescribed these. Patients we identified through record searches who wanted treatment for their depressive symptoms are also those for whom there might be clinical uncertainty. GPs could exclude anyone who was recruited through record searches.

Our finding that sertraline was not effective for depressive symptoms at 6 weeks is inconsistent with previous studies.^5^ Antidepressants could have an effect on depressive symptoms that is not large enough to be detected in a trial of this size. A meta-analysis by Cipriani and colleagues^5^ reported a standardised difference in means of −0·27 (95% CI −0·34 to −0·21) for sertraline versus placebo at a median follow-up of 8 weeks. This effect size is considerably larger than the −0·09 (−0·23 to 0·05) standardised difference in means from our trial at 6 weeks so the explanation of our trial being too small to detect the effect reported by Cipriani and colleagues^5^ seems unlikely. Our findings at 12 weeks of a −0·18 standardised mean difference (95% CI −0·33 to −0·03) are closer to the result reported by Cipriani and colleagues.^5^

Previous studies have used observer-rated scales to assess depression, such as the Hamilton Depression Scale.^30^ Unlike our self-reported scales, these measures allow interviewer discretion and increase the possibility of observer bias. Beneficial effects of sertraline on anxiety and self-reported improvement might therefore lead to a so-called halo effect, such that observers rate depressive symptoms as improved. There is evidence that observer rating can bias results of depression and anxiety assessments.^30^ The Hamilton Depression Scale also contains questions on the physical and psychological symptoms of generalised anxiety, which we found were affected by sertraline.

Our results suggest that the main benefits in the first 6 weeks of treatment with sertraline are on reduction of anxiety symptoms, such as worry and restlessness, rather than an improvement in depressive symptoms. Any effect on depressive symptoms takes longer to emerge and is more modest. However, an improvement in anxiety symptoms in someone presenting with depression could lead to a clinical benefit. In cases where there is uncertainty about prescribing an antidepressant, the presence of anxiety symptoms, such as worry and restlessness, could indicate an increased likelihood of benefit. Clinicians and patients should be aware of the symptoms that are likely to improve so that they can consider alternative management of depressive symptoms that might not respond.

Our findings support the prescription of SSRI antidepressants in a wider group of participants than previously thought, including those with mild to moderate symptoms who do not meet diagnostic criteria for depression or generalised anxiety disorder.

We did not find evidence that treatment response for either anxiety or depressive symptoms varied according to the severity of depressive or anxiety symptoms, even within this heterogenous group of participants. There could be subgroups within this population that did not benefit from the anxiety reducing effect; however, in the absence of any evidence for such subgroups we conclude that our results should be generalisable to the broad group of people included in our study.

In conclusion, our finding that sertraline affects anxiety more quickly than depressive symptoms has potential implications for understanding the mechanisms of antidepressant treatment. Depressive symptoms could take longer to reduce than anxiety symptoms and some of the reduction might be explained by the earlier effects on anxiety. Our results also challenge our reliance on placebo-controlled studies that have been done primarily for regulatory purposes. Investigation of pharmacological treatments after regulatory approval is needed. SSRIs are among the most commonly prescribed medications in the world and yet we still have an imperfect knowledge of their clinical effectiveness and indications for their use.

## Data sharing

De-identified individual participant data for the study, the study protocol, statistical analysis plan, and analytical code will be available to investigators for individual participant data meta-analyses that have been approved by independent review committees. Data will be available from the publication date of this Article, with no end date. Proposals for use of data and requests for access should be directed to glyn.lewis@ucl.ac.uk. To gain access, researchers will need to sign a data access agreement with the study sponsor (University College London, London, UK).
