# Supplementary material for: The clinical effectiveness of sertraline in primary care and the role of depression severity and duration (PANDA): a pragmatic, double-blind, placebo-controlled randomised trial
Source: Lancet Psychiatry. 2019 Nov;6(11):903–14. doi: 10.1016/S2215-0366(19)30366-9 (PMC7029306; doi:10.1016/S2215-0366(19)30366-9)
Supplement: Supplementary appendix [file mmc1.pdf]

THE LANCET
Psychiatry

Supplementary appendix

This appendix formed part of the original submission and has been peer reviewed. We post it as supplied by the authors.

Supplement to: Lewis G, Duffy L, Ades A, et al. The clinical effectiveness of sertraline in primary care and the role of depression severity and duration: a pragmatic, double-blind, placebo-controlled randomised trial. *Lancet Psychiatry* 2019; published online Sept 19. https://doi.org/10.1016/S2215-0366(19)30366-9.

Title: The clinical effectiveness of sertraline in primary care and the role of depression severity and duration: a pragmatic randomised controlled trial.

Running head: Antidepressants in primary care: the PANDA trial.

Supplementary Appendix

Authors

Gemma Lewis, PhD[1]
Larisa Duffy, BSc[1]
Anthony Ades, PhD[2]
Rebekah Amos, BSc[7]
Ricardo Araya, PhD[3]
Sally Brabyn, MSc[8]
Katherine S. Button, PhD[4]
Rachel Churchill, PhD[5]
Catherine Derrick, BSc[2]
Christopher Dowrick, MD[7]
Simon Gilbody, PhD[8]
Christopher Fawsitt, PhD[2]
William Hollingworth, PhD[2]
Vivien Jones, BA[2]
Tony Kendrick, MD[9]
David Kessler, PhD[2]
Daphne Kounali, PhD[2]
Naila Khan, PhD[7]
Paul Lanham, BA[1]
Jodi Pervin, BSc[8]
Tim J. Peters, PhD[2]
Derek Riozzie[7]
George Salaminios, MSc[1]
Laura Thomas, MSc[2]
Nicky J. Welton, PhD[2]
Nicola Wiles, PhD[2]
Rebecca Woodhouse, MSc[8]
Glyn Lewis, PhD[1]

Affiliations

[1]Division of Psychiatry, University College London, 6th Floor Maple House, 149 Tottenham Court Road, London W1T 7NF, UK.
[2]Bristol Medical School, University of Bristol, Oakfield House, Oakfield Grove, Bristol BS8 2BN, UK.
[3]Institute of Psychiatry, Psychology and Neuroscience, Kings' College London, Denmark Hill, London SE5 8AF, UK.
[4]Department of Psychology, University of Bath, 10 West, Bath BA2 7AT, UK.
[5]Centre for Reviews and Dissemination, University of York, Heslington, York YO10 5DD, UK.
[6]School of Nursing and Health Studies, University of Dundee, Airlie Place, Dundee DD1 4HJ, UK.
[7]Institute of Psychology Health and Society, University of Liverpool, Waterhouse Building Block B, Liverpool L69 3BX, UK.
[8]Department of Health Sciences, University of York, Seebohm Rowntree Building, Heslington, York YO10 5DD, UK.
[9]Primary Care and Population Sciences, Faculty of Medicine, University of Southampton, Aldermoor Health Centre, Southampton SO16 5ST, UK.
[10]Department of Sociology, Social Policy and Criminology, University of Liverpool, Eleanor Rathbone Building, Bedford Street South, Liverpool L69 7ZA, UK.

Correspondence to:

Dr Gemma Lewis, UCL Division of Psychiatry, 6th Floor, Maple House, 149 Tottenham Court Road, London W1T 7NF, gemma.lewis@ucl.ac.uk, 0203 108 7183.

Contents

Description	Page	Table
**Supplementary methods and results**		
Methods for statistical analyses that are reported in the Supplement	4	
Methods for post-hoc (unplanned) statistical analyses	4	
Description of post-hoc results including sub-group analyses and standardised differences in means	5	
**Supplementary Tables**		
Characteristics of GP practices	7	S1
Comparing the age and gender of the participants who were invited with those who participated	8	S2
Variables associated with missing data	9-10	S3
Adherence	11	S4
Adverse events	12	S5
Physical symptoms	12	S6
Analysis of continuous secondary outcomes adjusted for imbalance	13	S7
Analysis of binary secondary outcomes adjusted for imbalance	14	S8
Post-hoc analyses of physical health problems	15	S9
Baseline comparison of patients recruited through the mail-out and those recruited during GP consultation	16-17	S10
Post-hoc sub-group analyses of primary outcome (referral method, depression in the past, antidepressants in the past).	18	S11
Post-hoc sub-group analyses of primary outcome according to baseline severity	19	S12
Post-hoc sub-group analyses of primary outcome according to baseline duration	20	S13
Post-hoc sub-group analysis of anxiety symptoms according to baseline severity	21	S14
Post-hoc sub-group analysis of anxiety symptoms according to baseline duration	22	S15
Post-hoc analyses of response (>=50% reduction of the baseline score) as a secondary outcome	23	S16
Repeated measures analyses of PHQ-9 remission, with remission defined as scoring 0-4	24	S17
Changes to trial protocol version 3	25	
Trial protocol version 3	26-66	
Consort checklist	67-68	

Methods for statistical analyses reported in the Supplement

We described characteristics of the General Practice surgeries by reporting the percentage of practices with each characteristic. We compared the age and gender of the patients we invited to take part in the trial with those who actually participated by reporting the mean and SD for age and the number and percentage for gender (according to 'invited' versus 'participated').

To identify baseline demographic and clinical variables associated with missing data we made a binary variable at each follow-up time-point coded 0 if the PHQ score was not missing and 1 if the PHQ score was missing. We calculated odds ratios and 95% confidence intervals for each characteristic, at each follow-up, using univariable logistic regression models.

Methods for post-hoc (unplanned) analyses

We examined physical health comorbidities according to treatment allocation, in a descriptive analysis. We also compared the characteristics of patients recruited through GP consultation with those recruited through the record searches. We used a cut-off of <=4 for remission on the PHQ-9 and analysed this as a repeated measures secondary outcome. We conducted sub-group analyses to further investigate severity and duration. We also conducted analyses in which the outcome was absolute depression score (e.g. non-log transformed PHQ-9) and calculated interactions between treatment allocation and CIS-R depression severity score and duration. Finally we examined response as an outcome (50% reduction in depression score compared to baseline).

All sub-group analyses were conducted by running regression models stratified by the sub-group variable. We also report p values for interactions between each sub-group variable and

treatment allocation. For sub-group analyses according to the severity of depressive symptoms at baseline we use absolute (not log transformed) PHQ-9 or GAD-7 scores as the outcome, to investigate if mean rather than proportional differences varied by baseline severity.

We calculated logistic multilevel models using response (at least a 50% reduction in symptoms from baseline) across follow-up time-points as a binary outcome. This model was adjusted for PHQ and CISR depression severity scores at baseline and the stratification variables (severity assessed by CISR in three categories, duration in 2 categories and centre).

Results of post-hoc analyses

We found that 272 (42%) participants reported long-standing illness, disability or infirmity. Most prevalent were a mental health problem (21%), asthma or progressive lung disease (19%), diabetes (8%), arthritis (8%) and heart problems (4%). Prevalence of stroke, cancer or kidney disease was under 1% (Table S9). Patients recruited from GP consultations had more severe depressive and anxiety symptoms than those recruited through record searches, but were less likely to have taken antidepressants before (Table S10). We carried out sub-group analyses of the primary outcome and found no evidence that the treatment effect varied by method of recruitment or whether patients had used antidepressants or had depression in the past, Table S11. We examined other sub-groups defined by severity of depressive symptoms or ICD-10 diagnosis of depression. We found no evidence for a treatment effect on our primary outcome in any of these sub-groups, Table S12. There was also no evidence that the treatment effect varied according to depressive symptom duration, Table S13. We conducted similar analyses using GAD-7 scores as the outcome and found no evidence for an influence of severity or duration on the treatment effect, Tables S14 and S15.

We also analysed data using PHQ-9 and GAD-7 scores without log transforming and came to similar conclusions. The adjusted difference in PHQ-9 means between sertraline and placebo at 6 weeks was -0.51 (95% CI -1.33 to 0.31), equivalent to a standardised difference of -0.09 (95% CI -0.23 to 0.05). The adjusted difference in PHQ-9 means at 12 weeks was -1.07 (95% CI -1.96 to -0.19), a standardised difference of -0.19 (95% CI -0.33 to -0.03). We did not find any evidence that the treatment response varied with depression severity (p=0.89) or duration (p=0.73) in this model. The adjusted difference in GAD-7 means between sertraline and placebo at 6 weeks was -1.25 (-1.98 to -0.52), a standardised difference of -0.24 (95% -0.38 to -0.10). At 12 weeks this difference was -1.30 (95% CI -2.07 to -0.53), a standardised difference of -0.24 (95% CI -0.39 to -0.10). We found no evidence that treatment response varied with baseline severity (p=0.72) or duration (p=0.42) in this model.

The odds of response were 1.58 (95% CI 1.05 to 2.37, p=0.028) times higher in those taking sertraline compared to placebo, with a suggestion that this difference increased over time (p=0.062), Table S16. Evidence for an effect of sertraline on GAD-7 response was strong (adjusted odds ratio 2.51, 95% CI 1.58 to 4.00, p<.0001), with an indication that this became larger over time (p=0.094).

Table S1. Characteristics of the 179 General Practice surgeries used for recruitment

Characteristic	Percentage of practices with characteristic
Centre	
Bristol	31%
Liverpool	17%
London	40%
York	12%
Geographical location[a]	
Urban	86%
Rural	14%
List size[b]	
1-4999	12%
5000-9999	41%
10,000-14,999	24%
15,000+	23%
Number of GPs employed	
0-5	37%
6-10	41%
11-15	17%
16+	5%
Number of patients randomised	
0-4	74%
5-12	21%
13-20	4%
21+	1%
Index of Multiple Deprivation[c]	
1-3	20%
4-6	28%
7-10	52%

[a]Based on the 2011 rural-urban classification for output areas in England

[b]Number of patients enrolled in practice

[c]The Index of Multiple Deprivation combines UK national census information from 38 indicators into seven domains of deprivation (income; employment; health and disability; education, skills, and training; barriers to housing and services; living environment; and crime). This results in a deprivation score for each 32,482 'lower super output area' in England, geographical units used for the reporting of neighbourhood level statistics.

Table S2. Comparing the age and gender of the participants who were invited with those who participated.

	Age in years			Gender		
	Number	Mean	SD	Number	Female (n)	Female (%)
Trial participants[a]	653	39.7	15.0	653	384	61
Patients identified as potentially eligible and invited to participate[b]	11,636[c]	37.3	13.8	11,561[c]	7,001	59

[a]Included in the trial

[b]Identified as potentially eligible during the database search and sent an invitation letter. These data were provided by 53 of the 179 GP practices.

[c]A subset of the total number of participants who were identified as eligible and sent an invitation letter (31,645). This subset was comprised from the practices who returned these data.

Table S3. Univariable associations between baseline demographic and clinical variables and missing data on the PHQ-9 at each follow-up (binary outcome; 0=not missing, 1=missing). Odds ratio >1 indicates increased odds of missing data, <1 decreased odds.

Baseline variable	Odds ratio (95% confidence interval) p value at each follow-up		
	2 week (84/653, 13%, missing)	6 week (102/653, 16%, missing)	12 week (128/653, 20%, missing)
**Centre (n=653)**			
Bristol	**Ref**	**Ref**	**Ref**
Liverpool	0.87 (0.40 to 1.86) 0.715	0.76 (.38 to 1.52) 0.435	1.17 (0.65 to 2.11) 0.597
London	3.60 (2.09 to 6.22) <0.0001	3.36 (2.04 to 5.52) <0.0001	3.45 (2.14 to 5.56) <0.0001
York	0.60 (0.24 to 1.37) 0.227	0.37 (0.16 to 0.87) 0.022	0.68 (0.35 to 1.30) 0.242
**Age (n=653)**			
18-34	**Ref**	**Ref**	**Ref**
35-54	0.86 (0.53 to 1.40) 0.536	0.77 (0.48 to 1.21) 0.254	0.62 (0.40 to 0.94) 0.024
55-74	0.37 (0.17 to 0.81) 0.013	0.49 (0.25 to 0.94) 0.018	0.32 (0.17 to 0.61) <0.0001
**Sex (n=653)**			
Female	**Ref**	**Ref**	**Ref**
Male	1.38 (0.86 to 2.23) 0.185	1.10 (0.72 to 1.70) 0.567	1.21 (0.81 to 1.80) 0.344
**Ethnic (n=652)**			
White	**Ref**	**Ref**	**Ref**
Ethnic Minority	2.78 (1.55 to 4.98) 0.001	3.15 (1.83 to 5.42) <0.0001	2.88 (1.65 to 4.68) <0.0001
**Marital status (n=652)**			
Married or living as married	**Ref**	**Ref**	**Ref**
Single	1.72 (1.04 to 2.83) 0.035	1.95 (1.22 to 3.12) 0.007	2.08 (1.35 to 3.20) 0.001
Separated, divorced/ widowed	0.63 (0.26 to 1.49) 0.293	0.62 (0.28 to 1.40) 0.252	0.70 (0.34 to 1.43) 0.324
**Employment status (n=652)**			
In paid employment	**Ref**	**Ref**	**Ref**
Not employed	1.25 (0.78 to 2.01) 0.350	1.48 (0.96 to 2.27) 0.078	1.19 (0.79 to 1.78) 0.403
**Financial difficulty (n=652)**			
Comfortable or doing alright	Ref	Ref	Ref
Just about getting by	1.74 (1.05 to 2.92) 0.033	2.04 (1.27 to 3.27) 0.003	2.26 (1.4 to 3.48) <0.0001
Difficult or very difficult	2.39 (1.26 to 4.51) 0.007	2.70 (1.49 to 4.89) 0.001	2.88 (1.55 to 5.00) <0.0001
**Highest educational qualification (n=652)**			
A Level or higher	**Ref**	**Ref**	**Ref**
GCSE, standard grade/other	0.76 (0.43 to 1.33) 0.336	1.27 (0.78 to 2.06) 0.326	1.30 (0.84 to 2.01) 0.245
No formal qualification	1.14 (0.42 to 3.06) 0.797	2.67 (1.21 to 5.88) 0.015	2.31 (1.08 to 4.96) 0.031
**CIS-R total score (n=652)**			

0 to 11	**Ref**	**Ref**	**Ref**
12 to 20	1.29 (0.61 to 2.74) 0.502	0.81 (0.42 to 1.57) 0.527	0.84 (0.46 to 1.53) 0.572
20 to 49	1.59 (0.82 to 3.09) 0.172	1.17 (0.67 to 2.04) 0.593	1.14 (0.68 to 1.90) 0.628
**CIS-R depression duration (n=652)**			
Less than 2 years	**Ref**	**Ref**	**Ref**
2 years or more	1.16 (0.72 to 1.88) 0.538	0.83 (0.52 to 1.32) 0.432	0.92 (0.61 to 1.39) 0.683
**ICD-10 CIS-R depression diagnosis (n=652)**			
No	**Ref**	**Ref**	**Ref**
Yes	1.23 (0.80 to 1.90) 0.335	1.20 (0.75 to 1.91) 0.444	1.23 (0.83 to 1.82) 0.294
**ICD-10 CIS-R anxiety diagnosis (n=652)**			
No	Ref	Ref	Ref
Yes	1.16 (0.76 to 1.77) 0.486	1.51 (0.95 to 2.39) 0.081	1.18 (0.80 to 1.74) 0.395
**Depression in the past (n=652)**			
No	**Ref**	**Ref**	**Ref**
Yes	0.83 (0.50 to 1.38) 0.473	0.91 (0.51 to 1.58) 0.714	0.97 (0.60 to 1.57) 0.906
**Antidepressant in the past (n=652)**			
No	**Ref**	**Ref**	**Ref**
Yes	0.78 (0.51 to 1.20) 0.256	0.63 (0.40 to 0.99) 0.047	0.83 (0.56 to 1.22) 0.338
**PHQ total score (n=651)**	1.04 (1.00 to 1.08) 0.050	1.02 (0.99 to 1.07) 0.223	1.05 (1.02 to 1.09) 0.002
**CIS-R depression severity score (n=652)**	1.03 (0.98 to 1.08) 0.205	1.02 (0.98 to 1.07) 0.339	1.04 (0.99 to 1.08) 0.082
**BDI-II total score (n=652)**	1.03 (1.01 to 1.05) 0.006	1.02 (1.00 to 1.05) 0.032	1.03 (1.01 to 1.05) 0.002
**Social support score (n=652)**	1.03 (0.98 to 1.09) 0.166	0.97 (0.92 to 1.03) 0.326	0.98 (0.93 to 1.03) 0.334
**Number of life events in past 6 months (n=652)**	0.70 (0.60 to 0.83) <0.0001	1.25 (1.05 to 1.50) 0.013	1.40 (1.20 to 1.63) <.0001
**GAD-7 score (n=652)**	1.05 (1.01 to 1.10) 0.011	1.04 (1.00 to 1.09) 0.050	1.06 (1.02 to 1.10) 0.002
**SF-12 mental health subscale (n=649)**	0.99 (0.97 to 1.01) 0.178	0.98 (0.96 to 1.01) 0.157	0.98 (0.96 to 1.00) 0.050
**SF-12 physical health subscale (n=649)**	0.98 (0.96 to 1.00) 0.045	1.00 (0.98 to 1.03) 0.774	0.99 (0.97 to 1.01) 0.394

Table S4: Adherence to medication and beliefs about medication according to treatment allocation 2, 6 and 12 weeks after randomisation.

Questionnaire item/s	2 weeks, n (%)		P value[c]	6 weeks, n (%)		P value[c]	12 weeks, n (%)		P value[c]
	Placebo	Sertraline		Placebo	Sertraline		Placebo	Sertraline	
>=80% adherence[a]	262 (97)	241 (96)	0.376	245 (89)	219 (85)	0.149	211 (82)	204 (80)	0.608
Have started taking medication[b]	272 (93)	251 (90)	0.128	278 (98)	259 (97)	0.689	261 (98)	258 (97)	0.987
Currently taking medication[b]	269 (99)	243 (97)	0.188	264 (95)	239 (93)	0.262	222 (85)	204 (80)	0.109
Taken their tablets every day or taken nearly all their tablets[b]	268 (99)	243 (97)	0.190	267 (97)	242 (94)	0.108	243 (94)	240 (94)	0.973
Thought they were taking sertraline	30 (11)	74 (31)	<0.0001	52 (19)	115 (46)	<0.0001	42 (17)	123 (50)	<0.0001

[a]We used a five-item self-report adherence scale developed for the CoBalt study (reference 29 in main manuscript). >=80% adherence is a binary variable, with a score of 0 (range 0–4) indicating at least 80% adherence.

[b]Additional adherence items we included in the questionnaires

[c]Calculated using Pearson's chi-squared test

Table S5. Serious adverse events

Allocation	Brief description of event	SAE	Seriousness [a]	Related to IMP	Outcome [b]
Sertraline	Suicidal ideation	Yes	6	Possibly	1
Placebo	Hospitalised for physical illness	Yes	3	Not related	1
Sertraline	Non-cardiac chest pain	Yes	3	Unlikely	1

Abbreviations: IMP - Investigational medicinal product; SAE – serious adverse event

[a]Seriousness: 1=Resulted in Death, 2=life Threatening, 3=required inpatient or prolonged existing hospitalisation, 4=resulted in persistent or significant disability/incapacity, 5=resulted in congenital anomaly/birth defect, 6= Important Medical Event.

[b]Outcome: 1= Resolved, 2 = Resolved with sequelae, 3 = Unresolved, 4= Worsening, 5 = Fatal, 6= not assessable.

Table S6. Physical symptoms that are potential SSRI side-effects[a] according to treatment allocation 2, 6 and 12 weeks after randomisation.

	Mean (SD) number of symptoms		P	Mean (SD) frequency of symptoms		P
	Placebo	Sertraline		Placebo	Sertraline	
2 weeks	7.68 (4.85)	7.98 (4.54)	0.4513	39.38 (9.06)	40.09 (9.07)	0.3504
6 weeks	7.10 (5.00)	7.41 (4.63)	0.4546	38.45 (8.83)	39.06 (8.61)	0.4175
12 weeks	6.77 (5.27)	6.78 (4.86)	0.9863	48.77 (17.98)	49.09 (17.32)	0.8360

[a] Recorded with a modified Toronto scale.

Table S7. Repeated measures analyses of continuous secondary outcomes at 2, 6, 12 weeks, adjusted for variables not balanced at baseline.

Outcome	Sertraline		Placebo		Adjusted proportional difference[a] (95% CI)	P value
PHQ-9 (n=547)	n	Mean (SD)	n	Mean (SD)		
2 weeks	277	9.94 (5.83)	292	10.32 (5.55)	.96 (.87 to 1.07)	
6 weeks	266	7.98 (5.63)	284	8.76 (5.86)	.95 (.86 to 1.06)	
12 weeks	262	6.90 (5.83)	263	8.02 (6.12)	.87 (.78 to .97)	
Average over time					.93 (.86 to 1.01)	.075
Group by time interaction						.093
BDI-II (n=540)						
2 weeks	273	18.77 (11.08)	286	19.10 (11.17)	.98 (.89 to 1.10)	
6 weeks	266	14.82 (10.44)	285	15.91 (10.74)	.95 (.85 to 1.07)	
12 weeks	256	12.44 (10.96)	259	14.78 (11.70)	.84 (.74 to .94)	
Average over time					.92 (.84 to 1.02)	.10
Group by time interaction						.015
GAD-7 (n=546)						
2 weeks	277	7.55 (5.49)	291	8.16 (5.26)	.91 (.81 to 1.02)	
6 weeks	264	5.55 (5.19)	284	6.96 (5.24)	.78 (.70 to .88)	
12 weeks	263	4.95 (5.30)	263	6.27 (5.28)	.76 (.68 to .86)	
Average over time					.82 (.74 to .90)	<.0001
Group by time interaction						.008
Outcome	Sertraline		Placebo		Adjusted mean difference (95% CI)	P value
SF-12 Mental Health (n=538)	n	Mean (SD)	n	Mean (SD)		
2 weeks	275	37.32 (11.47)	291	35.37 (11.36)	1.67 (-.01 to 3.44)	
6 weeks	254	41.95 (12.35)	277	38.67 (11.91)	2.97 (1.24 to 4.70)	
12 weeks	263	42.70 (12.91)	264	39.71 (11.87)	2.91 (1.18 to 4.64)	
Average over time					2.49 (1.21 to 3.77)	<.0001
Group by time interaction						.22
SF-12 Physical Health (n=538)						
2 weeks	275	51.92 (9.18)	291	52.40 (6.64)	-.69 (-1.74 to .36)	
6 weeks	245	51.98 (8.39)	277	51.76 (9.90)	-.35 (-1.42 to .73)	
12 weeks	263	51.92 (8.53)	264	52.50 (9.99)	-.88 (-1.96 to .20)	
Average over time					-.64 (-1.47 to .18)	.13
Group by time interaction						.78

[a]These models use log transformed scores as the outcome. Adjusted proportional differences can be interpreted as the difference in scores between randomised groups expressed as a proportion (or percentage). Models are adjusted for baseline measure of each outcome (continuous), baseline CIS-R depression severity score and stratification variables (baseline total CIS-R score in three categories; duration of depressive episode in two categories; site) and variables not balanced at baseline (sex, ICD-10 depression diagnosis, marital status).

Table S8. Repeated measures analyses of binary secondary outcomes at 2, 6, 12 weeks, adjusted for variables not balanced at baseline.

Outcome	Sertraline		Placebo		Adjusted[a] odds ratio (95% CI)	P value
PHQ-9 remission[b] (n=547)	n	n (%)	n	n (%)		
2 weeks	277	145 (52)	292	136 (47)	1.42 (.80 to 2.51)	
6 weeks	267	169 (63)	285	164 (58)	1.36 (.76 to 2.43)	
12 weeks	262	190 (73)	263	170 (65)	1.83 (.99 to 3.42)	
Average over time					1.50 (.98 to 2.32)	.063
Group by time interaction						.49
BDI-II remission[b] (n=541)						
2 weeks	273	58 (21)	286	58 (20)	1.06 (.54 to 2.08)	
6 weeks	266	94 (35)	285	91 (32)	1.32 (.73 to 2.44)	
12 weeks	256	131 (51)	259	102 (39)	2.74 (1.49 to 5.05)	
Average over time					1.63 (1.04 to 2.56)	.34
Group by time interaction						.014
Feeling better (n=546)						
2 weeks	279	110 (39)	292	89 (30)	1.66 (1.07 to 2.57)	
6 weeks	267	157 (59)	285	132 (46)	1.92 (1.25 to 2.94)	
12 weeks	264	156 (59)	265	112 (42)	2.46 (1.57 to 3.82)	
Average over time					1.98 (1.47 to 2.66)	<.0001
Group by time interaction						.16

[a]All multi-level models adjusted for baseline measure of each outcome (continuous), baseline CIS-R depression severity score, stratification variables (baseline total CIS-R score in three categories; duration of depressive episode in two categories; site) and variables not balanced at baseline (sex, ICD-10 depression diagnosis, marital status).
[b]Remission defined as a score <=10.

Table S9. Physical health problems reported at baseline in the sample overall and according to treatment allocation

Physical health problem[a]	Overall (N=653)	Sertraline (n=324)	Placebo (n=329)
Any	272 (42%)	140 (43%)	132 (40%)
Diabetes	23 (8%)	11 (48%)	12 (52%)
Asthma or COPD[b]	52 (19%)	29 (56%)	23 (44%)
Arthritis	21 (8%)	12 (57%)	9 (43%)
Heart disease or heart problem	11 (4%)	4 (36%)	7 (64%)
Stroke	2 (0.7%)	1 (50%)	1 (50%)
Cancer	3 (1%)	2 (67%)	1 (33%)
Kidney disease	4 (1%)	1 (25%)	3 (75%)
None of the above	98 (36%)	54 (55%)	44 (45%)

[a]Assessed with the CIS-R

[b]Chronic Obstructive Pulmonary Disease (COPD) includes progressive lung diseases: emphysema, chronic bronchitis, and refractory (non-reversible) asthma.

Table S10. Baseline comparison of patients recruited through the GP record search and those recruited during GP consultation.

Characteristic	Recruitment method	
	Record search (n=466)	Consultation (n=187)
Site[a]		
Bristol	133 (29%)	132 (71%)
Liverpool	114 (25%)	2 (1%)
London	90 (19%)	52 (28%)
York	129 (28%)	1 (0.5%)
CIS-R total score[a]		
0 to 11	107 (23%)	22 (12%)
12 to 20	128 (27%)	45 (24%)
20 to 49	231 (50%)	120 (64%)
CIS-R depression duration[a]		
Less than 2 years	293 (63%)	146 (78%)
2 years or more	173 (37%)	41 (22%)
Age		
18-34	188 (40%)	78 (42%)
35-54	181 (39%)	78 (42%)
55-74	97 (21%)	31 (17%)
Sex		
Female	286 (61%)	98 (52%)
Male	180 (39%)	89 (48%)
ICD-10 CIS-R depression diagnosis[b]		
Yes	232 (50%)	123 (66%)
No	233 (50%)	64 (34%)
ICD-10 CIS-R anxiety diagnosis[b]		
Yes	192 (41%)	107 (57%)
No	273 (59%)	80 (43%)
Ethnic group[b]		
White	423 (91%)	156 (83%)
Ethnic minority	42 (9%)	31 (17%)
Marital status[b]		
Married or living as married	172 (37%)	83 (44%)
Single	213 (46%)	83 (44%)
Separated, divorced or widowed	80 (17%)	21 (11%)
Employment status[b]		
In paid employment	304 (65%)	129 (69%)
Not employed	161 (35%)	58 (31%)
Financial difficulty[b]		
Living comfortably or doing alright	267 (57%)	97 (52%)
Just about getting by	144 (31%)	60 (32%)
Finding it difficult or very difficult	54 (12%)	30 (16%)
Highest educational qualification[b]		
A Level or higher	331 (71%)	119 (64%)
GCSE, standard grade or other	114 (25%)	55 (29%)
No formal qualification	20 (4%)	13 (7%)
Depression in the past[b]		
Yes	403 (87%)	119 (64%)

No	62 (13%)	68 (20%)
Antidepressant in the past[b]		
Yes	319 (69%)	72 (39%)
No	146 (31%)	115 (62%)
PHQ-9 total score (range 0-27)	11.31 (5.76)	13.71 (5.55)
CIS-R total score (range 0-64)	19.91 (9.97)	24.56 (9.83)
CIS-R depression severity score (range 0-21)[c]	10.09 (4.91)	11.80 (4.66)
BDI-II total score (range 0-63)	24.01 (10.54)	23.87 (10.07)
GAD-7 score (range 0-21)	8.70 (5.08)	11.25 (5.32)
Social support score (range 1-24)	12.57 (3.81)	12.84 (3.85)
SF-12 mental health subscale (range 0-100)	35.56 (11.20)	29.76 (10.16)
SF-12 physical health subscale (range 0-100)	52.27 (9.45)	51.58 (10.32)
Number of life events in past 6 months	1.11 (1.12)	1.50 (1.32)
Number of physical symptoms in past 2 weeks	9.67 (5.31)	11.05 (5.59)
Frequency of physical symptoms (range 55-112)[d]	42.94 (10.19)	46.32 (12.35)

Data are n (%) or mean (SD).
CIS-R data are missing for one person.
Ranges for continuous scales are possible rather than actual ranges
PHQ-9=Patient Health Questionnaire, 9-item version
CIS-R=Clinical Interview Schedule Revised
BDI-II=Beck Depression Inventory, second edition
GAD-7=Generalised Anxiety Disorder Assessment, 7-item version
SF-12= Short-Form Health Survey

[a]The total CIS-R score assesses severity of symptoms of common mental disorder. Total CIS-R score in three categories was a stratification variable at randomisation: 0-11 (minimal symptoms); 12-19 (moderate to severe symptoms); 20+ (severe symptoms)

[b]A CIS-R diagnosis uses the criteria and threshold required to meet an ICD-10 clinical diagnosis of depression or anxiety. CIS-R data missing for one person

[c]The CIS-R depression severity score (range 0-21) assesses the severity of depressive symptoms

[d] How often during the past two weeks the patient experienced each symptom: 1 (not at all); 2 (several days); 3 (more than half the days); 4 (nearly every day)

Table S11. Post-hoc sub-group analyses of primary outcome (log transformed PHQ9 scores at 6 weeks). Means are for non-log transformed PHQ9 scores at 6 weeks.

Recruitment	Placebo		Sertraline		Comparison
	n	Mean (SD)	n	Mean (SD)	Adjusted proportional difference (95% CI)*
Record search	202	8.76 (5.93)	203	7.95 (5.69)	0.97 (0.85 to 1.10)
GP consultation	83	8.73 (5.69)	64	8.20 (5.51)	0.90 (0.73 to 1.11)
P value for interaction: 0.592					
Depression in the past	Placebo		Sertraline		Comparison
	n	Mean (SD)	n	Mean (SD)	Adjusted proportional difference (95% CI)*
Depressed in past	227	8.93 (6.07)	217	8.14 (5.61)	0.96 (0.84 to 1.08)
Not depressed in past	58	8.07 (4.89)	49	7.29 (5.74)	0.92 (0.72 to 1.19)
P value for interaction: 0.86					
Antidepressants in past	Placebo		Sertraline		Comparison
	n	Mean (SD)	n	Mean (SD)	Adjusted proportional difference (95% CI)*
Yes	175	8.98 (5.99)	160	7.97 (5.54)	0.96 (0.84 to 1,08)
No	110	8.38 (5.63)	106	8.00 (5.79)	0.99 (0.83 to 1.17)
P value for interaction: 0.21					

*Primary analysis model: Adjusted for baseline PHQ-9 score, continuous baseline CIS-R depression score and the stratification variables (baseline total CIS-R score in three categories; duration of depressive episode in two categories and site).

Table S12. Post-hoc sub-group analyses of PHQ-9 scores at 6 weeks, according to severity of depressive symptoms at baseline. Models use absolute (non-log transformed) PHQ-9 scores as outcome, to examine whether absolute differences differ according to baseline severity.

Baseline measure of depression severity	Placebo		Sertraline		Mean difference (95% CI)*
	n (%)	Mean (SD)	n (%)	Mean (SD)	
No ICD-10 depression diagnosis	125 (44)	6.76 (5.20)	129 (49)	6.20 (4.89)	-.44 (-1.53 to .65)
ICD-10 depression diagnosis	159 (56)	10.34 (5.89)	137 (52)	9.66 (5.78)	-.72 (-1.96 to .51)
P value for interaction: 0.792					
	Placebo		Sertraline		Mean difference (95% CI)*
CIS-R total score	n (%)	Mean (SD)	n (%)	Mean (SD)	
0 to 11	52 (18)	4.40 (4.12)	56 (21)	4.48 (3.90)	-.14 (-1.499 to 1.22)
12 to 20	79 (28)	7.70 (5.54)	72 (27)	6.81 (4.73)	-.66 (-2.27 to .95)
20 to 49	153 (54)	10.80 (5.59)	138 (52)	10.01 (5.81)	-.56 (-1.80 to .68)
P value for interaction: 0.733					
	Placebo		Sertraline		Mean difference (95% CI)*
	n (%)	Mean (SD)	n (%)	Mean (SD)	
CIS-R total score<28	203 (71)	7.89 (5.40)	198 (74)	6.91 (5.22)	-.85 (-1.76 to .05)
CIS-R total score>=28	81 (29)	10.96 (6.39)	68 (26)	11.10 (5.65)	.56 (-1.25 to 2.37)
P value for interaction: 0.153					
	Placebo		Sertraline		Mean difference (95% CI)*
	n (%)	Mean (SD)	n (%)	Mean (SD)	
CIS-R depression score<15	215 (76)	7.74 (5.24)	212 (80)	6.98 (5.20)	-.66 (-1.54 to .22)
CIS-R depression score>=15	69 (24)	11.96 (6.55)	54 (20)	11.91 (5.60)	-.05 (-2.14 to 2.04)
P value for interaction: 0.397					
	Placebo		Sertraline		Mean difference (95% CI)*
	n (%)	Mean (SD)	n (%)	Mean (SD)	
PHQ9 score <10	101 (36)	5.16 (4.11)	114 (43)	5.27 (4.28)	-.75 (-1.94 to .44)
PHQ9 score >=10	183 (64)	10.75 (5.74)	152 (57)	10.01 (5.68)	-.06 (-1.11 to .98)
P value for interaction: 0.440					
	Placebo		Sertraline		Mean difference (95% CI)*
	n (%)	Mean (SD)	n (%)	Mean (SD)	
PHQ9 score <20	253 (89)	8.44 (5.59)	242 (91)	7.50 (5.25)	-.77 (-1.59 to .06)
PHQ9 score >=20	31 (11)	11.42 (7.28)	24 (9)	12.83 (7.06)	1.86 (-1.69 to 5.41)
P value for interaction: 0.071					
	Placebo		Sertraline		Mean difference (95% CI)*
	n (%)	Mean (SD)	n (%)	Mean (SD)	
BDI-II score <10	16 (6)	2.44 (2.69)	20 (8)	5.00 (4.35)	.88 (-2.15 to 3.91)
BDI-II score >=10	268 (94)	9.14 (5.78)	246 (92)	8.22 (5.66)	-.67 (-1.53 to .20)
P value for interaction: 0.197					
	Placebo		Sertraline		Mean difference (95% CI)*
	n (%)	Mean (SD)	n (%)	Mean (SD)	
BDI-II score <29	199 (70)	7.50 (5.36)	187 (70)	6.44 (4.95)	-.77 (-1.69 to .15)
BDI-II score >=29	85 (30)	11.72 (5.95)	79 (30)	11.62 (5.50)	.19 (-1.51 to 1.88)
P value for interaction: 0.314					

*The placebo group was the reference category and negative mean differences indicate lower scores in the sertraline compared to placebo group. All models adjusted for baseline PHQ9 score, continuous baseline CIS-R depression score and stratification variables (baseline total CIS-R score in three categories; duration of depressive episode in two categories; centre).

All interaction terms and between treatment allocation and the severity variable used to create the sub-group.

Table S13. Post-hoc sub-group analyses of primary outcome (log transformed PHQ9 scores at 6 weeks), stratified according to the duration of the depressive episode at baseline.

Baseline measure of depression duration	Placebo		Sertraline		Adjusted proportional difference (95% CI)*
	n (%)	Mean (SD)	n (%)	Mean (SD)	
Less than two years	187 (66)	8.15 (5.84)	179 (67)	7.72 (5.58)	0.97 (0.84 to 1.11)
Two years or more	97 (34)	9.95 (5.75)	87 (33)	8.53 (5.73)	0.90 (0.75 to 1.09)
P value for interaction: 0.690					
Baseline measure of depression duration	Placebo		Sertraline		Adjusted proportional difference (95% CI)*
	n (%)	Mean (SD)	n (%)	Mean (SD)	
Less than 6 months	64 (12)	7.66 (5.15)	54 (12)	6.96 (5.78)	0.94 (0.74 to 1.20)
6 months to 1 year	41 (23)	9.80 (5.91)	48 (20)	9.46 (5.27)	1.00 (0.74 to 1.34)
Between 1 and 2 years	48 (14)	9.44 (6.40)	44 (18)	9.50 (5.41)	0.90 (0.70 to 1.16)
Between 2 and 5 years	48 (17)	10.08 (5.76)	50 (17)	8.58 (5.33)	0.88 (0.69 to 1.12)
More than 5 years	49 (17)	9.82 (5.80)	37 (14)	8.46 (6.31)	0.95 (0.70 to 1.28)
P value for interaction: 0.734					

*All models were adjusted for baseline PHQ9 score, continuous baseline CIS-R depression score and stratification variables (baseline total CIS-R score in three categories; duration of depressive episode in two categories; centre).

All interaction terms are between treatment allocation and the duration variable used to create the sub-group.

Table S14. Post-hoc sub-group analyses of GAD7 scores at 6 weeks, according to severity of depressive and anxiety symptoms at baseline. Models use absolute (non-log transformed) GAD-7 scores as the outcome, to examine whether mean differences vary according to baseline severity.

Baseline measure of depression severity	Placebo		Sertraline		Mean difference (95% CI)*
	n (%)	Mean (SD)	n (%)	Mean (SD)	
No ICD-10 depression diagnosis	124 (44)	5.25 (4.59)	127 (49)	4.36 (4.59)	-0.87 (-1.86 to .13)
ICD-10 depression diagnosis	159 (56)	8.33 (5.34)	136 (51)	6.60 (5.47)	-1.68 (-2.76 to -0.16)
P value for interaction: 0.27					
	Placebo		Sertraline		Mean difference (95% CI)*
	n (%)	Mean (SD)	n (%)	Mean (SD)	
No ICD-10 GAD diagnosis	151 (53)	5.42 (4.67)	150 (57)	4.09 (4.19)	-1.31 (-2.18 to -.42)
ICD-10 GAD diagnosis	133 (47)	8.71 (5.33)	113 (43)	7.42 (5.74)	-1.10 (-2.33 to .14)
P value for interaction: 0.89					
	Placebo		Sertraline		Mean difference (95% CI)*
CIS-R total score	n (%)	Mean (SD)	n (%)	Mean (SD)	
0 to 11	52 (18)	2.91 (3.22)	56 (21)	2.36 (2.94)	-.79 (-1.88 to .31)
12 to 20	79 (28)	5.37 (4.50)	69 (26)	4.24 (3.71)	-1.14 (-2.46 to .18)
20 to 49	152 (54)	9.28 (5.06)	138 (52)	7.43 (5.69)	-1.47 (-2.62 to -.32)
P value for interaction: 0.39					
	Placebo		Sertraline		Mean difference (95% CI)*
	n (%)	Mean (SD)	n (%)	Mean (SD)	
CIS-R total score<28	202 (71)	5.82 (4.65)	195 (74)	4.23 (4.26)	-1.52 (-2.30 to -.73)
CIS-R total score>=28	81 (29)	9.88 (5.54)	68 (26)	9.21 (5.79)	-.35 (-2.06 to 1.35)
P value for interaction: 0.23					
	Placebo		Sertraline		Mean difference (95% CI)*
	n (%)	Mean (SD)	n (%)	Mean (SD)	
CIS-R depression score<15	214 (53)	6.04 (4.67)	209 (47)	4.57 (4.51)	-1.41 (-2.19 to -.64)
CIS-R depression score>=15	69 (47)	9.88 (5.86)	54 (53)	9.15 (6.01)	-.63 (-2.55 to 1.39)
P value for interaction: 0.78					
	Placebo		Sertraline		Mean difference (95% CI)*
	n (%)	Mean (SD)	n (%)	Mean (SD)	
PHQ9 score <10	101(36)	4.22 (3.92)	112 (43)	3.26 (3.65)	-1.33 (-2.40 to -.26)
PHQ9 score >=10	182 (64)	8.51 (5.27)	151 (57)	7.19 (5.51)	-1.00 (-1.92 to -.07)
P value for interaction: 0.34					
	Placebo		Sertraline		Mean difference (95% CI)*
	n (%)	Mean (SD)	n (%)	Mean (SD)	
PHQ9 score <20	252 (89)	6.61 (5.00)	239 (91)	5.04 (4.77)	-1.50 (-2.24 to -.76)
PHQ9 score >=20	31 (11)	9.97 (6.22)	24 (9)	10.25 (6.21)	1.71 (-1.34 to 4.77)
P value for interaction: 0.047					
	Placebo		Sertraline		Mean difference (95% CI)*
	n (%)	Mean (SD)	n (%)	Mean (SD)	
GAD-7 score <8	118 (42)	4.07 (3.58)	118 (45)	3.32 (3.92)	-.82 (-1.73 to .09)
GAD-7 score >=8	166 (58)	9.02 (5.27)	146 (55)	7.36 (5.40)	-1.55 (-2.65 to -.45)
P value for interaction: 0.31					
	Placebo		Sertraline		Mean difference (95% CI)*
	n (%)	Mean (SD)	n (%)	Mean (SD)	
GAD-7 score <14	221 (78)	5.67 (4.36)	205 (78)	4.40 (4.26)	-1.09 (-1.84 to -.35)
GAD-7 score >=14	63 (22)	11.51 (5.58)	58 (22)	9.48 (6.15)	-1.50 (-3.55 to .56)
P value for interaction: 0.42					

*The placebo group was the reference category and negative mean differences indicate lower scores in the sertraline compared to placebo group. All models adjusted for baseline PHQ9 score, continuous baseline CIS-R depression score and stratification variables (baseline total CIS-R score in three categories; duration of depressive episode in two categories; centre).

All interaction terms and between treatment allocation and the severity variable used to create the sub-group.

Table S15. Post-hoc sub-group analyses of GAD-7 scores at 6 weeks (log transformed), according to the duration of anxiety symptoms at baseline.

Baseline measure of anxiety duration	Placebo		Sertraline		Adjusted proportional difference (95% CI)*
	n (%)	Mean (SD)	n (%)	Mean (SD)	
Less than two years	194 (68)	6.12 (4.93)	183 (69)	4.73 (4.59)	.79 (.69 to .93)
Two years or more	90 (32)	8.77 (5.33)	81 (31)	7.40 (5.98)	.75 (.60 to .95)
P value for interaction: 0.74					
Baseline measure of anxiety duration	Placebo		Sertraline		Adjusted proportional difference (95% CI)*
	n (%)	Mean (SD)	n (%)	Mean (SD)	
Less than 6 months	69 (24)	6.80 (4.86)	50 (19)	5.18 (4.86)	.79 (.59 to 1.16)
6 months to 1 year	25 (9)	6.52 (4.46)	31 (12)	5.87 (4.34)	1.04 (.72 to 1.00)
Between 1 and 2 years	33 (12)	6.79 (5.28)	44 (17)	5.34 (4.96)	.77 (.54 to 1.15)
Between 2 and 5 years	54 (19)	9.06 (5.30)	41 (16)	7.46 (5.75)	.72 (.52 to 1.51)
More than 5 years	36 (13)	8.36 (5.73)	40 (15)	7.33 (6.27)	.82 (.59 to 1.05)
P value for interaction: 0.95					
Baseline measure of depression duration	Placebo		Sertraline		Adjusted proportional difference (95% CI)*
	n (%)	Mean (SD)	n (%)	Mean (SD)	
Less than two years	194 (68)	6.12 (4.93)	183 (69)	4.73 (4.59)	.77 (.66 to .91)
Two years or more	90 (32)	8.77 (5.33)	81 (31)	7.40 (5.98)	.78 (.63 to .97)
P value for interaction: 0.42					
Baseline measure of depression duration	Placebo		Sertraline		Adjusted proportional difference (95% CI)*
	n (%)	Mean (SD)	n (%)	Mean (SD)	
Less than 6 months	69 (24)	6.80 (4.86)	50 (19)	5.18 (4.86)	.69 (.51 to .91)
6 months to 1 year	25 (9)	6.52 (4.46)	31 (12)	5.87 (4.34)	1.08 (.78 to 1.51)
Between 1 and 2 years	33 (12)	6.79 (5.28)	44 (17)	5.34 (4.96)	.58 (.44 to .79)
Between 2 and 5 years	54 (19)	9.06 (5.30)	41 (16)	7.46 (5.75)	.76 (.58 to .98)
More than 5 years	36 (13)	8.36 (5.73)	40 (15)	7.33 (6.27)	.72 (.50 to 1.04)
P value for interaction: 0.96					

*All models were adjusted for baseline PHQ9 score, continuous baseline CIS-R depression score and stratification variables (baseline total CIS-R score in three categories; duration of depressive episode in two categories; centre).

All interaction terms are between treatment allocation and the duration variable used to create the sub-group.

Table S16. Repeated measures analyses of binary response outcome at 2, 6, 12 weeks.

Outcome	Sertraline		Placebo		Adjusted[a] odds ratio (95% CI)	P value
PHQ-9 response[b] (n=547)	n	n (%)	n	n (%)		
2 weeks	277	145 (52)	292	136 (47)	1.12 (.60 to 2.07)	
6 weeks	267	169 (63)	285	164 (58)	1.47 (.86 to 2.51)	
12 weeks	262	190 (73)	263	170 (65)	2.16 (1.26 to 3.72)	
Average over time					1.58 (1.05 to 2.37)	.028
Group by time interaction						.062
BDI-II response[b] (n=541)						
2 weeks	273	58 (21)	286	58 (20)	0.81 (0.41 to 1.58)	
6 weeks	266	94 (35)	285	91 (32)	1.33 (0.72 to 2.45)	
12 weeks	256	131 (51)	259	102 (39)	2.35 (1.26 to 4.39)	
Average over time					1.42 (0.88 to 2.23)	.15
Group by time interaction						.0005
GAD-7 response[b] (n=546)						
2 weeks	278	83 (30)	291	72 (25)	1.44 (0.78 to 2.69)	
6 weeks	265	135 (51)	284	89 (31)	3.97 (2.14 to 7.32)	
12 weeks	264	152 (58)	263	112 (42)	2.68 (1.46 to 4.93)	
Average over time					2.51 (1.58 to 4.00)	<.0001
Group by time interaction						.094

[a]All multi-level models adjusted for baseline measure of each outcome (continuous), baseline CIS-R depression severity score, stratification variables (baseline total CIS-R score in three categories; duration of depressive episode in two categories; site) and variables not balanced at baseline (sex, ICD-10 depression diagnosis, marital status).
[b]Defined as a 50% reduction in symptoms or greater, from baseline.

Table S17. Repeated measures analyses of PHQ-9 remission, with remission defined as scoring 0-4.

Outcome	Sertraline		Placebo		Adjusted[a] odds ratio (95% CI)	P value
PHQ-9 remission	n	n (%)	n	n (%)		
2 weeks	277	56 (20)	292	44 (15)	1.63 (.78 to 3.44)	
6 weeks	267	92 (34)	285	80 (28)	1.50 (.78 to 2.89)	
12 weeks	262	117 (45)	263	92 (35)	2.16 (1.13 to 4.13)	
Average over time					1.63 (1.07 to 2.47)	.022
Group by time interaction						.44

[a]Adjusted for baseline measure of each outcome (continuous), baseline CIS-R depression severity score, and stratification variables (baseline total CIS-R score in three categories; duration of depressive episode in two categories; site.

Changes to protocol version 3 (protocol version 3 was dated 30/05/2014 and was the last version of the protocol before the study started 01/01/2015):

1. To capture patient-rated change, we added the measure of self-reported improvement as a secondary outcome in protocol version 4 dated 05/03/2015 and approval of this change was received from the ethics committee (NRES) 15/05/2015.
2. It was apparent towards the later stages of designing the RCT and in formulating the detailed analysis plan (uploaded before any analyses were performed to: http://discovery.ucl.ac.uk/10041458/ and approved by the Trial Steering Committee), that we would have insufficient statistical power to estimate plausible interaction effects. Our power calculation and primary analysis (as stated in the analysis plan: http://discovery.ucl.ac.uk/10041458/) were therefore based on a primary aim to examine the clinical effectiveness of sertraline versus placebo. This change was made in protocol version 4, dated 05/03/2015.
3. In line with the change above (point 2), we changed the primary analysis to a linear regression of log transformed PHQ-9 scores at 6 weeks. Interactions between severity and duration at baseline and treatment response were planned as exploratory. This is documented in the detailed analysis plan (uploaded before any analyses were performed to: http://discovery.ucl.ac.uk/10041458/ and approved by the Trial Steering Committee)
4. Due to a poor response rate from GP mail-outs, the recruitment process was modified to include a further telephone call to non-responders. This change was submitted in protocol version 5 dated 16/11/2015 and approval was received from NRES 01/12/2015.
5. Due to a release of SmPC v7 (the information about the drug that the manufacturing company releases that includes all the safety and adverse effects) that mentions an increased QT interval associated with Sertraline and after discussions with our Sponsor, we amended the protocol. This change was submitted in protocol version 6 dated 11/02/2016 and approval was received from NRES 04/04/2016.
6. We updated the GP referral sheet and GP eligibility confirmation to reflect the change referenced in point 4 above. This change was submitted in protocol version 6 dated 11/02/2016 and approval was received from NRES 04/04/2016.
7. The sertraline patient information leaflet was replaced with the updated version, issued by Bristol Labs Ltd. This change was submitted in protocol version 6 dated 11/02/2016 and approval was received from NRES 04/04/2016.
8. Minor amendments were made to include nurse prescribers, change the procedure and contact details for reporting Pharmacovigilance and add ‘GP practices’ to the insurance section. This change was submitted in protocol version 6.1 dated 22/04/2016 and approval was received from the trial sponsor, 13/07/2016. This was a minor change which required approval from the sponsor rather than the ethics committee.
9. Attrition was higher than expected and a minor amendment was submitted to recruit more participants than originally intended. This change to the protocol was submitted in protocol version 6.1 dated 22/04/2016 and approval was received from the trial sponsor, dated 13/07/2016.
10. Results from analyses of the EQ5D and emotional processing secondary outcomes will be reported in a separate paper. The EQ5D will form part of the economics analysis and the emotional processing tasks will be analysed using complex computational modelling.
11. Qualitative analyses of the PANDA RCT that were planned to aid recruitment were not conducted because recruitment rates were higher than expected.

panda
what are the indications for Prescribing ANtiDepressAnts that will lead to a clinical benefit?

**Full title of trial**	What are the indications for Prescribing ANtiDepressAnts that will lead to a clinical benefit?<br><br>A Phase IV, double-blind randomised placebo-controlled, parallel group multi-site trial; of sertraline compared to placebo in patients presenting with depressive symptoms in primary care where treatment with SSRIs is uncertain
**Short title**	PANDA
**Version and date of protocol**	Version 3 30/05/2014
**Sponsor:**	University College London (UCL)
**Sponsor protocol number**	13/0413
**Funder (s) :**	NIHR
**EudraCT no**	2013-003440-22
**ACTIVE IMP(s):**	Sertraline
**PLACEBO IMP(s):**	Placebo capsules
**Phase of trial**	Phase IV
**Sites(s)**	Multisite

UCL

Chief investigator:
Glyn Lewis
Professor of Psychiatric Epidemiology
Mental Health Sciences Unit
University College London
67-73 Riding House St
London W1W 7EJ
glyn.lewis@ucl.ac.uk
+44 (0) 207 679 9711

Sponsor Representative:
Kirsty Adams
Joint Research Office, 1st Floor Maple House, Suite A
149 Tottenham Court Road,
London W1T 7NF.

Postal address:
Joint Research Office, UCL Gower Street, London WC1E 6BT

Signatures

The Chief Investigator and the JRO have discussed this protocol. The investigators agree to perform the investigations and to abide by this protocol

The investigator agrees to conduct the trial in compliance with the approved protocol, EU GCP and UK Regulations for CTIMPs (SI 2004/1031; as amended), the UK Data Protection Act (1998), the Trust Information Governance Policy (or other local equivalent), the Research Governance Framework (2005' 2nd Edition; as amended), the Sponsor's SOPs, and other regulatory requirements as amended.

Chief investigator

Prof Glyn Lewis		30/05/2014
UCL	Signature	Date

Sponsor Representative

Dr Nick McNally		
UCL	Signature	Date

Contents

Signatures 28
Contents 29
List of abbreviations 31
1 Trial personnel 34
2 Summary 34
3 Introduction 36
3.1 Background 36
3.2 Preclinical data 36
3.3 Clinical data 36
3.4 Rationale and risks/benefits 37
3.5 Assessment and management of risk 38
4 Objectives 39
5 Trial design 39
5.1 Overall design 39
6 Selection of Subjects 40
6.1 Inclusion criteria 40
6.2 Exclusion criteria 40
7 Recruitment 40
8 Study procedures and schedule of assessments 41
8.1 Informed consent procedure 41
8.2 Randomisation procedures 42
8.3 Unblinding 42
8.4 Screening Period 43
8.5 Baseline assessments 43
8.6 Treatment procedures 43
8.7 Subsequent assessments 43
Flowchart of study assessments 45
8.8 Methods 45
8.9 Definition of end of trial 45
8.10 Discontinuation/withdrawal of participants and ‘stopping rules’ 45
9 Name and description of all drugs used in the trial 46
9.1 Treatment of subjects 46

9.2 Concomitant medication ... 46
10 Investigational Medicinal Product ... 47
10.1 Name of IMP ... 47
10.2 Summary of findings from non-clinical studies ... 47
10.3 Summary of findings from clinical studies ... 47
10.4 Summary of known and potential risks and benefits ... 47
10.5 Description and justification of route of administration and dosage ... 53
10.6 Dosages, dosage modifications and method of administration ... 53
10.7 Preparation and labelling of Investigational Medicinal Product ... 53
10.8 IMP accountability ... 53
10.10 Source of IMPs including placebo ... 53
10.11 Dose modifications ... 54
10.12 Assessment of compliance ... 54
10.13 Post-trial IMP arrangements ... 54
11 Recording and reporting the adverse events and reactions ... 54
11.1 Definitions ... 54
11.2 Recording adverse events ... 55
11.4 Procedures for recording and reporting Serious Adverse Events ... 57
11.4.1 Notification of deaths ... 57
11.4.2 Reporting SUSARs ... 57
11.4.3 Multisite responsibilities ... 57
11.4.4 Development Safety Update Reports ... 57
11.4.5 Annual progress reports ... 57
11.4.6 Pregnancy ... 58
11.4.6 Overdose ... 58
11.4.7 Reporting Urgent Safety Measures ... 58
11.5 The type and duration of the follow-up of subjects after adverse events. 58
11.5.1 Notification of Serious Breaches to GCP and/or the protocol (SPON/S15) 59
12 Data management and quality assurance ... 59
12.1 Confidentiality ... 59
12.2 Data collection tools and source document identification ... 59
12.3 Data handling and analysis ... 60
13 Record keeping and archiving ... 60

14 Statistical Considerations ... 60
14.1 Outcomes ... 60
14.1.1 Primary outcome ... 60
14.1.2 Secondary outcomes ... 60
14.2 Sample size and recruitment ... 61
14.2.1 Sample size calculation ... 61
14.2.2 Planned recruitment rate ... 61
14.3 Statistical analysis plan ... 62
14.3.1 Summary of baseline data and flow of patients ... 62
14.3.2 Primary outcome analysis ... 62
14.3.3 Secondary outcome analysis ... 62
14.3.4 Sensitivity and other planned analyses ... 62
14.4 Randomisation methods ... 62
14.5 Interim analysis ... 62
14.6 Other statistical considerations ... 63
15 Name of Committees involved in trial ... 63
16 Direct Access to Source Data/Documents ... 63
17 Ethics and regulatory requirements ... 63
18 Monitoring requirement for the trial ... 63
19 Finance ... 64
20 Insurance ... 64
21 Publication policy ... 64
22 Statement of compliance ... 64
23 References ... 65

List of abbreviations

AE	Adverse Event
AR	Adverse Reaction
CA	Competent Authority
CI	Chief Investigator
CRF	Case Report Form
CRO	Contract Research Organisation

CTA	Clinical Trial Authorisation
CTIMP	Clinical Trial of Investigational Medicinal Product
DMC	Data Monitoring Committee
DSUR	Development Safety Update Report
EC	European Commission
EMEA	European Medicines Agency
EU	European Union
EUCTD	European Clinical Trials Directive
EudraCT	European Clinical Trials Database
EudraVIGILANCE	European database for Pharmacovigilance
GAfREC	Governance Arrangements for NHS Research Ethics
GCP	Good Clinical Practice
GMP	Good Manufacturing Practice
ICF	Informed Consent Form
IDMC	Independent Data Monitoring Committee
IMP	Investigational Medicinal Product
IMPD	Investigational Medicinal Product Dossier
ISF	Investigator Site File
ISRCTN	International Standard Randomised
MA	Marketing Authorisation
MHRA	Medicines and Healthcare products Regulatory Agency
MS	Member State
Main REC	Main Research Ethics Committee
NHS R&D	National Health Service Research & Development
PI	Principal Investigator
PIS	Participant Information Sheet
QA	Quality Assurance
QC	Quality Control
QP	Qualified Person for release of trial drug
RCT	Randomised Control Trial
REC	Research Ethics Committee
SAR	Serious Adverse Reaction
SAE	Serious Adverse Event
SDV	Source Document Verification

SOP	Standard Operating Procedure
SmPC	Summary of Product Characteristics
SSA	Site Specific Assessment
SUSAR	Suspected Unexpected Serious Adverse Reaction
TMG	Trial Management Group
TSC	Trial Steering Committee

1 Trial personnel

Chief Investigator (CI) Glyn Lewis

Professor of Psychiatric Epidemiology

Mental Health Sciences Unit

University College London

67-73 Riding House St, London W1W 7EJ

E-mail: glyn.lewis@ucl.ac.uk

tel: 0207 6799711

Sponsor's representative Kirsty Adams

Joint Research Office, 1st Floor Maple House, Suite A

149 Tottenham Court Road, London W1T 7NF.

Postal address:

Joint Research Office, UCL Gower Street, London WC1E 6BT

e-mail: gemma.jones@ucl.ac.uk
tel: 020 7679 6502 fax: 020 3108 2312

PRIMENT Clinical Trials Unit Joanne Palmer

UCL Medical School Royal Free Campus, London, NW3 2PF

Tel 02077940 500 ext 36715

Email: joanne.palmer@ucl.ac.uk

Statistician Prof Tim Peters

School of Clinical Sciences

University of Bristol, St Michael's Hill, Bristol BS2 2DZ
e-mail: tim.peters@bristol.ac.uk
tel: 0117 331 1833 fax: 0117 331 1698

Trial Manager Larisa Duffy

University of Bristol

Oakfield House, Oakfield Grove, Bristol, BS8 2BN

Email: l.duffy@bristol.ac.uk

Tel: 01173313348

2 Summary

Title: **What are the indications for Prescribing ANtiDepressAnts that will lead to a clinical benefit?**

A Phase IV, double-blind randomised placebo-controlled, parallel group multi-site trial; of Sertraline compared to placebo in patients presenting

	with depressive symptoms in primary care where treatment with SSRIs is uncertain
**Short title:**	**PANDA**
**Trial medication:**	Sertraline vs. Placebo
**Phase of trial:**	Phase IV
**Objectives:**	The primary objective is to investigate the severity and duration of the depressive symptoms that are associated with a clinically important response (compared to placebo) to sertraline in people with depression.<br><br>The secondary objective is to investigate quality of life, the economic cost and whether performances on emotional processing tasks are associated with response to treatment with sertraline.
**Type of trial:**	Phase IV, double-blind, randomised, parallel group, multi-site trial in patients presenting in depressive symptoms in primary care.
**Trial design and methods:**	Patients presenting with depression in primary care where there is ambiguity whether SSRI treatment would be prescribed will be individually randomised between sertraline and placebo. 50mg sertraline capsules at one capsule daily will be administered for one week increasing to two capsules daily. If the patient does not respond then the PI will decide whether the dose should be increased to 3 capsules. Patients will receive IMP for 12 weeks in total. The primary outcome will be the PHQ9 questionnaire that measures depressive symptoms at 6 weeks. The 12 week outcome will be a secondary outcome to look for more persistent effects. During the recruitment phase we will also carry out qualitative interviews with participants and those refusing to take part in order to inform our recruitment methods.
**Trial duration per participant:**	12 weeks plus 2 weeks for tapering medication.
**Estimated total trial duration:**	30 months
**Planned trial sites:**	Bristol, York, Liverpool, Southampton, London
**Total number of participants planned:**	683
**Main inclusion/exclusion criteria:**	1) Uncertainty of GP and patient about the possible benefits of antidepressants (any severity and duration of depression may be included).<br><br>2) aged 18 to 74 years (inclusive)<br><br>3) have <u>not</u> been treated with antidepressants in the previous 8 weeks<br>4) people who are having other interventions such as low intensity IAPT can also take part<br>***Exclusion criteria:***<br>1) have other psychiatric disorders, i.e. bipolar disorder, eating disorder or psychosis<br>2) have major alcohol or substance abuse problem/s<br>3) are not able to complete the study questionnaires, inc registered blind<br>4) women who are currently pregnant or planning pregnancy or lactating<br>5) people taking contraindicated medications: monoamine oxidase inhibitors and pimozide.<br>6) severe hepatic impairment

	7) people with bleeding disorders such as haemophilia, Christmas disease and von Willebrands disease, as well as those with past medical history of bleeding gastric or duodenal ulcers or other significant bleeding disorders
**Statistical methodology and analysis:**	The CONSORT guidelines will be followed and an analysis plan will be agreed with the Trial Steering Committee and Independent Data Monitoring and Ethics Committee. Our primary outcome will be the PHQ9 at 6 weeks. Baseline comparability will also be described. The primary analysis will use a linear regression model to estimate interaction terms between the randomised treatment and baseline severity of symptoms and baseline duration of symptoms. We will adjust for baseline scores on the PHQ9 and will have two strata of severity and two of duration. We will adopt an intention to treat approach and carry out analyses to investigate the likely impact of any missing data using multiple imputation.<br><br>*Economic analysis:* NHS and Personal Social Services resource use will be valued using local and, where available, national unit cost data. The cost-effectiveness analysis will evaluate the efficiency of sertraline stratified by baseline severity and duration of symptoms. Net monetary benefit (NMB) of treatment for individual patients will be calculated, based on standard NICE willingness to pay thresholds.

3 Introduction

3.1 Background

Depression is a common condition that affects between 2% and 3% of the population at any one time. Depression is commonly treated with antidepressant medication.(1) In England and Wales there were 47m prescriptions for antidepressants in 2011 with a substantial cost to the NHS. Selective serotonin reuptake inhibitors (SSRIs) are the first line antidepressant recommended by NICE guidelines.(2) Many people consult with depressive symptoms and general practitioners (GPs) often feel under pressure to provide some treatment.(3) Some people with depression will recover spontaneously and it is not clear at present which people will benefit from a course of antidepressants. Furthermore it is not known whether the current diagnostic criteria for depression as described in ICD10 or DSM5 indicate benefit from antidepressants. Depression is also difficult to assess accurately during a short primary care consultation. As a result, general practitioners (GPs) often have to make a difficult decision about whether an individual will benefit from an SSRI.

3.2 Preclinical data

A summary of the pre-clinical data may be found in the Summary of Product Characteristics.

3.3 Clinical data

Sertraline is a selective serotonin reuptake inhibitor (SSRI) that is licensed for the treatment of depression and has a well-established efficacy profile. As a result it is one of the recommended SSRIs to use as a first choice in the treatment of depression.(2, 4) It is very widely prescribed in primary and secondary care in the UK and elsewhere in the world. There are about 500,000 prescriptions a month in England at present. A

recent network meta-analysis suggested that, if anything, it was more efficacious and better tolerated than most of the other SSRIs.(5)

The safety profile is good and it is well tolerated. The commonest adverse effects tend to be nausea and sedation. Along with other SSRIs there has been concern about the possibility of an early effect on “jitteriness” as a result of increased serotonin activity with all SSRIs.(6) However, there is little high quality empirical evidence to support this syndrome and little consistency about how it is defined. Sertraline is also much safer than the older tricyclic antidepressants if taken in overdose.(7)

The effectiveness of antidepressants for milder depression is reviewed below. Much of this evidence is not concerned with sertraline but with other SSRIs and tricyclic antidepressants. Tricyclic antidepressants often but not always have serotonin reuptake inhibition activity so we think these results will also apply to sertraline.

3.4 Rationale and risks/benefits

This study is designed to refine the indications for the use of antidepressants in people with depression. At present it is not known whether all people who meet the diagnostic criteria for depression will benefit from an antidepressant compared to a placebo. There is evidence that the more severe the symptoms of depression the more likely someone is to benefit. Kirsch(8) and Khan(9) have carried out systematic reviews of aggregate data and provided evidence that severity at baseline is related to outcome. We are aware of two individual patient data meta-analyses by Fournier (10) who found evidence for a severity relationship and Gibbons (11) in a much larger study found no evidence of a relationship with severity. A systematic review of studies of depression that is “minor” or does not meet the criteria for depression found no evidence for a treatment effect of antidepressants. (12)

It is also possible that people who do not meet the diagnostic criteria may benefit. For example, evidence suggests that antidepressants are effective for people with dysthymia(13) so the NICE guidelines recommend SSRIs for “persistent subthreshold depressive symptoms” but gives no guidance on the duration of the persistence. We are proposing, as adopted by current UK guidelines(2, 14) that symptom severity and duration of symptoms are two separate dimensions that might both help to predict response to antidepressants. The primary aim of the study is therefore to test these hypotheses concerned with severity and duration. This will then lead to improved guidance for clinicians and patients about when antidepressants are likely to benefit.

There are also psychological treatments for depression such as cognitive behaviour therapy and collaborative care. However, these are usually thought of as complementing the pharmacological treatment so the optimal use of antidepressants is still important even if people will also receive psychological treatments. The evidence to date suggests that the treatment effects of psychological and pharmacological treatments are additive.(15)

Our aim is therefore to carry out a randomised controlled trial in order to investigate the severity and duration of depressive symptoms that are associated with a clinically important response to sertraline in people with depression. We plan to assess severity and duration using a standardised assessment that can then be used to guide prescription in primary care. The inclusion criteria are pragmatic and broad to reflect the current dilemma in clinical practice. Older ages, over 74 years, are not included as additional clinical issues concerned with cognitive decline and social care become more common after that age.

The benefits will be in improved guidance and thereby increasing the likelihood that a prescription will benefit and reduce prescriptions that are not needed.

We will include patients presenting in primary care aged 18-74 with depressive symptoms and both the GP and patient are unsure whether there will be significant clinical benefit from taking SSRI antidepressants and not currently on antidepressants (or in previous 8 weeks). We want to keep the inclusion criteria pragmatic and broad to reflect the current dilemma in clinical practice.(29) We therefore think that the uncertainty of GP

and patient about the possible benefits of antidepressants is the key entry criterion for the trial. We will not impose any additional criteria of severity and duration ourselves.

Choice of antidepressant: Sertraline is the second most commonly prescribed SSRI in the UK. It is no longer under patent (28 tab pack £1.39). There are few pharmacological differences between the SSRIs and so matters such as convenience and lack of drug interactions are important in determining choice of antidepressant. Though citalopram is more commonly prescribed there have been recent concerns expressed about the possibility of increasing the QT interval on the ECG. Therefore we have chosen to use sertraline.

3.5 Assessment and management of risk

People with depression have an increased risk of self-harm and suicide. We will therefore have a self-harm SOP in place and staff will be trained to follow this procedure.

Sertraline will be provided in 50mg capsules. Patients will start treatment at 50 mg daily to ensure that they can tolerate the dose. The usual dose in primary care is 100mg and we will recommend that all participants take 2 capsules (100mg) following the first week of treatment unless they cannot tolerate the increased dose. After 4 weeks from the start of taking study medication, the participants can increase to 3 capsules if they have not responded and with the agreement of the PI. It is usual to start on a lower dose in order to reduce early adverse effects.

The exclusions listed above include the elderly, contraindicated medication, pregnancy and severe hepatic impairment. These are regarded as those who might be most at risk from the medication so this risk will be eliminated. We will ask the patients' GP to confirm in writing the absence of any medical conditions for exclusion before they will be considered as eligible. tThe participants will be explained about the possible risks in pregnancy. We will also perform a urine pregnancy test on all women of child bearing potential. There will also be discussion regarding the use of effective means of contraception throughout the trial (such as hormonal or barrier method of birth control) We will also establish that adequate contraceptive practice will be in place for the trial period. The general practitioners will continue their usual medical supervision of the participants and will be asked to inform the trial team of any serious adverse events experienced. The PI or delegate will be available to answer any medical questions raised by the general practitioner or any other doctor who is involved with the participants. There will be a 24 hour telephone number for medical emergencies and to enable unblinding.

A withdrawal syndrome has been described after the abrupt stopping of SSRI medication. The NICE guidelines recommend tapering over about a 4 week period but this is primarily for other SSRIs and venlafaxine that have more marked withdrawal symptoms. We have therefore recommended that participants taper over 2 weeks, during which time they should consult with their general practitioner in order to discuss further treatment.

The research assessments will also include a questionnaire that will list a selection of the main adverse effects (AEs of special interest) that are associated with sertraline as by using a questionnaire the research team will have a more accurate and less biased account of the adverse events (AEs). We will also ask an open ended question about adverse events to capture any SAEs and the listed AEs of special interest.

Sertraline is very commonly prescribed for this indication therefore we regard the risk as low and akin to normal clinical care. However this trial has been classified as Type B rather than A because of the presence of the placebo arm.

The potential risk of those on placebo is that they might in principle have benefited from the sertraline and their depression will worsen. If the patient is deteriorating to a degree that creates clinical concern they will be advised to stop the trial medication and consult the general practitioner for further management. The GP will be provided with the unblinded treatment allocation in order to inform further care. The other risk of placebo is if the participant is allergic to the placebo and excipients. In that case they will be excluded.

There is also the risk of self-poisoning with the IMP. This risk and the management of this is described in more detail in section 11.4.6.

4 Objectives

Primary: To investigate the severity and duration of the depressive symptoms that are associated with a clinically important response (compared to placebo) to sertraline in people with depression.

Secondary: to investigate quality of life, the economic costs and whether emotional processing tasks are associated with response to treatment with sertraline.

5 Trial design

5.1 Overall design

Randomised, double blind placebo controlled study in which eligible participants are randomised to sertraline or placebo. The sertraline will be encapsulated and matching placebo capsules produced in order to maintain the blind during the study.

Trial treatment will be for 12 weeks with assessments at 2, 6 and 12 weeks. The main treatment response compared to placebo occurs within about 6 weeks. We also want to obtain an early account of adverse events and clinical response at 2 weeks as the first signs of improvement can occur at that point.(16) The 12 week assessment will provide evidence for any sustained benefit.

The participants will be asked to take one capsule per day (50mg sertraline or placebo) and after one week to increase this to two capsules per day as long as they do not experience any side effects. This information will be collected by the local PI or delegate and recorded in the trial medication questionnaire. If the patient is not responding they will be asked to consult their general practitioner who will in turn consult the PI who will make the decision to increase the medication to 3 capsules if appropriate. Following the 12 week assessment the patients will be unblinded (see section 8.3), patients will be supplied with additional IMP supply for up to 2 weeks and will be instructed to taper their dose until they are able to attend their GP to receive their treatment allocation and discuss further treatment.

Qualitative interviews to investigate recruitment:

In order to help guide the recruitment strategy we will carry out interviews with participating and declining participants and GPs while piloting our trial protocol. The aim will be to inform the recruitment strategy by exploring the acceptability of the study design to participants and GPs. Thirty participants will be interviewed or until there are no new themes emerging. Interviews will either be face-to-face (at participants home, GP practice or University premises) or via telephone and would be semi-structured, guided by a topic guide developed from literature. Participants will be interviewed after 12 weeks participation in the trial.

We will also identify participants who have refused to take part in the study. We will ask an additional question during the recruitment procedures to ask participants if they agree to explain at more length why they do not want to take part. Those who give consent will have a brief interview over the telephone to give their reasons for not participating.

We will invite all participating GP's to take part in a brief 15 minute 'feed-back' telephone interview, to explore what can be improved in the recruitment process during the early stages of the trial.

The interviews described above would be tape recorded and transcribed. Participants would be invited to take part in these interviews. The consent procedure for the trial would describe this process and ask for separate consent for these qualitative interviews.

6 Selection of Subjects

We will allow hypnotic medication and other non-pharmacological treatment options including low intensity psychosocial treatments as provided by IAPT or counselling. We will record this information and can investigate any impact on the findings in secondary analyses (and also use for the economic analysis).

There is marked comorbidity between depression and anxiety disorders. The GP will refer patients as per the exclusions and inclusions listed below. They will exclude any patients with anxiety disorders that they wish to treat with SSRIs as there will not be clinical equipoise about the benefits of SSRI medication. We will assess anxiety disorders at baseline and any influence of comorbid anxiety (that we expect will be quite common) on outcome can be examined in exploratory analyses.

6.1 Inclusion criteria

1. Age: 18-74 (inclusive)
2. Gender: both female and male
3. Depression presenting in primary care
4. Clinical equipoise about the benefits of SSRI medication

6.2 Exclusion criteria

1. Antidepressant medication in the preceding 8 weeks.
2. Unable to read, understand and/or complete questionnaires
3. Other psychiatric disorders: psychosis, schizophrenia, bipolar disorder, mania, hypomania, dementia, eating disorder.
4. Major alcohol or substance misuse problems
5. Currently on contraindicated medication: monoamine oxidase Inhibitors within the past 14 days or pimozide.
6. Patients with poorly controlled epilepsy
7. Known allergies to the IMP, placebo or excipients
8. Concurrent enrolment in another IMP trial
9. Women who are currently pregnant or planning pregnancy or lactating
10. Severe hepatic impairment
11. Bleeding disorders such as such as haemophilia, Christmas disease and von Willebrands disease, as well as those with past medical history of bleeding gastric or duodenal ulcers or other significant bleeding disorders

7 Recruitment

The potential participants will be recruited to a trial site by referral from GPs who will see patients either at consultation or perform a database search.

At consultation. The GP will be asked to refer potential participants by fax (or secure email) to the local trial centre giving the patients' permission for further contact if eligible. The researcher will contact the patient to confirm eligibility for the trial and arrange the baseline assessment visit.

Database searches. We will also ask GPs or their administrative staff to carry out record searches according to a Record Search SOP to identify people in whom the GP has recorded low mood, depression symptoms and are not currently on antidepressants. We will ask the GP to write to these individuals so they can consider joining the study.

Before entry into the trial the GP will record and then fax the research team a sheet that confirms that the potential participant does not have any disorders or medication that would exclude them.

8 Study procedures and schedule of assessments

8.1 Informed consent procedure

The potential participants will be posted or given in person by their GP a patient information sheet (PIS) that gives details of the study. Following a screening telephone call, they will be invited to a baseline assessment; at least 24 hours will be given for consideration of the PIS. Both at the screening telephone call and at this meeting the study will be explained, including the aims, methods, anticipated benefits and potential hazards by a research assistant who will be GCP trained, suitably qualified, experienced and delegated this duty by the CI/PI on the appropriate delegation log. The patients will be given the opportunity to ask questions. Patients will be under no obligation to enter the trial and they will be advised that they can withdraw at any time during the trial without providing a reason. The local PI or delegated physician will be available by phone if there are any queries. Adequate time will be given for consideration by the patient; it is expected that most patients will be willing to consent to the study at the baseline visit. If a patient is unsure then they may be rescheduled for consent and baseline assessments at a later date. A delegated member of staff will then obtain written consent. Consent will not denote enrolment into the trial. A copy of the consent form will be given to the patient, the original will be retained in the trial file and a copy will be sent to the general practitioner. A letter will also be sent to the patient's GP to inform them of their enrolment in the trial. No clinical trial procedures will be conducted prior to taking consent.

If new safety information results in significant changes in the risk/benefit assessment, the participant information sheet and consent form will be reviewed and updated if necessary. All subjects, including those already being treated, will be informed of any new information, giving a copy of the revised information sheet and subjects will be re-consented as appropriate.

Qualitative Interview consent procedure

Participants who have refused to take part in the study will be identified. An additional question will be asked during the recruitment procedures to ask participants if they agree to explain at more length why they do not want to take part. Those who give their permission will have a brief interview over the telephone to give their reasons for not participating.

In the early phases of the trial, participants who consent to take part in the RCT will be invited to take part in qualitative interviews about the recruitment procedure and processes of the trial. The PIS will describe these interviews and the consent form will allow participants to give separate consent for these interviews if they also want to take part in this element.

8.2 Randomisation procedures

The randomisation will be conducted by PRIMENT CTU using a remote computer generated code (Sealed Envelope). The randomisation will be stratified by severity and duration of depression and centre and with random block lengths, the randomisation list will be held by Sealed Envelope. The delegated member of staff will access a web based interface using a unique user name and password. The delegated member of staff will enter the unique study identification number for the participant and the stratification variables. The random treatment allocation will then be sent to the pharmacy. Blinded confirmation of randomisation will be printed and filed.

8.3 Unblinding

Patients will be provided with a contact card so that treating clinicians who may be external to the study team can be unblinded to information on treatment in any clinical emergency. If unblinding is required a formal request will be made to an authorised member of the study team (through the contact number provided on the 24 hour contact card) who will access sealed envelope to reveal the treatment allocation.

If the CI/investigating team require the breaking of the code they will have access to sealed envelope in order to unblind the patient.

Study codes should only be broken for valid medical or safety reasons e.g. in the case of an SAE where it is necessary for the treating professional to know which treatment the patient is receiving before they can treat the patient. Where possible for treating professionals outside the research team, the unblinding request will be discussed with the investigating team (CI, PI or delegate) so that a formal assessment can be undertaken.

If in the opinion of the treating physician the code must be broken immediately then this must be undertaken without further assessment. The treating physician will treat the medical emergency as appropriate upon receipt of the treatment allocation.

The CI/PI or delegate will document the breaking of the code and reasons for doing so on the CRF / data collection tool, in the site file and medical notes. Code breaks will also be documented at the end of the study in any final study report and or statistical report.

The CI/Investigating team will notify the JRO (acting on behalf of the sponsor) in writing as soon as possible following the code break detailing the necessity of the code break. The CI will also notify relevant authorities and trial committees (in accordance with their charter). Where possible, members of the research team should remain unblinded.

When participants have ended the study and their outcome data has been entered into the database they can request to be told their treatment allocation to placebo or active medication. This information will be provided to their GP so the participant will need to consult their GP and any further treatment can be discussed at that consultation. The trial team will remain blind to this information. Further details will be in the Unblinding SOP.

The DSMC can request unblinded data.

The following procedure will be used to unblind for the submission of a SUSAR report to the regulatory agencies.

- An authorized user from the JRO will access Sealed Envelope to obtain the treatment allocation for a specified patient.
- This information will not be forwarded to the trial team and kept in the JRO Sponsor file.

8.4 Screening Period

The GPs will refer potential patients who they feel meet the eligibility criteria and either obtain the agreement of potential participants for them to be contacted by the study team or write to them. All potential patients will be listed on a screening log. The study team will confirm eligibility prior to the baseline assessment and before the consent process is started.

8.5 Baseline assessments

See section 8.1 for consent process to be undertaken at this visit.

All eligibility criteria will be assessed prior to the baseline visit , on confirmation of eligibility the patient will be entered into the trial. There is no maximum duration between the patient giving permission for us to contact them and the baseline visit.

Baseline: self-administered computerised CISR,(17, 18); PHQ9,(19) BDI-II,(20) SF12, EQ5D,(21) GAD7,(22). Emotional (face recognition and word tasks) processing tasks(23) that are sensitive to antidepressants and may be a marker of treatment response; questions about the belief in the efficacy of antidepressants.

Medical History

Concomitant Medication

Randomisation will be completed as per 8.2.

Instructions for how to take the IMP and 24 hour emergency contact card will be given.

IMP dosing will start when the patient collects the medication from the general practice.

8.6 Treatment procedures

Intervention: Participants will be provided with encapsulated sertraline 50mg tablets or an identical placebo. They will take one capsule (Sertraline 50mg or placebo) once daily increasing to two capsules once daily after a week if tolerated. If the patients reduce to one capsule once daily they can again try to take 2 capsules once daily at any point in the trial. If participants do not respond they can consult their general practitioner who will then contact the PI who will increase the medication to 3 capsules if appropriate. Patients will be treated for 12 weeks and then a 2 week tapering period following the 12 week assessment. See section 10.0 for further details.

8.7 Subsequent assessments

All subsequent interviews will either be conducted face-to-face or over the telephone. The researcher will aim to conducted the follow ups at the 2, 6 and 12 weeks, however there will be occasions when this may not be possible and the details of how this will be conducted will be part of a Follow-up SOP The participants will continue to be invited to interview unless they have withdrawn from the trial. The date of the interview will be recorded and the analysis plan will include measures to investigate the timing of the follow up appointments. They will be continued to be followed up even if they have stopped taking the study medication.

2 weeks PHQ9, BDI-II, SF12, EQ5D, GAD7, Emotional processing tasks, modified Morisky adherence measure(24), and pill count, side effects of antidepressant a modified version of the Toronto Side Effects scale as used in GENPOD.(25), open ended question about adverse events, concomitant medication

6 weeks: PHQ9, BDI-II, SF12, EQ5D, GAD7, Emotional processing tasks, modified Morisky adherence measure and pill count. side effects of antidepressant a modified version of the Toronto Side Effects scale as used in GENPOD. open ended question about adverse events, concomitant medication.

12 weeks: PHQ9, BDI-II, SF12, EQ5D, GAD7, modified Morisky adherence measure and pill count.(18). side effects of antidepressant a modified version of the Toronto Side Effects scale as used in GENPOD.(25) open ended question about adverse events, concomitant medication, health service and other resource use.

After 12 weeks: recording of resource use from GP electronic health record for the economic analysis.

Methods for the Qualitative study are described in section 5.1

Flowchart of study assessments

	Screening	Baseline				
	Telephone and GP information		Week 2	Week 6	Week 12	Week 12 +2
Informed Consent		X				
Medical History		X				
Eligibility determination, incl pregnancy test at BL	X	X				
Symptom, quality of life		X	X	X	X	
Emotional processing tasks		X	X	X		
Randomisation		X				
IMP administration		X	X	X	X	X
IMP dispensing		X		X		
Adverse Events review			X	X	X	
Concomitant Medication review			X	X	X	
Pill count and IMP compliance check				X	X	
unblinding on request						X

8.8 Methods

See Section 12.2 for data collection tools

8.9 Definition of end of trial

The end of the trial for the participant will be after the 12 week, when data has been collected and entered onto the database. They will be supplied with 2 weeks study medication after this point and instructions on how to taper the medication. This will also give the participant an opportunity to consult their GP about any further treatment.

The end of trial is defined as collection of the last data regarding resource use.

8.10 Discontinuation/withdrawal of participants and 'stopping rules'

Subjects may be discontinued at any time but once dosing has occurred every attempt should be made to continue assessments to ensure the safety of the subject. Specific reasons for discontinuing a subject may be:

- Voluntary discontinuation by the subject who is at any time free to discontinue his participation in the study, without prejudice to further treatment
- Risk to subjects as judged by the investigator
- Severe non-compliance to protocol as judged by the investigator.
- Incorrectly randomised subjects.

- Adverse Events

A subject that discontinues will always be asked about the reason(s) for discontinuation and the presence of any adverse events. If possible, they will be seen and assessed by an investigator(s) or delegate. Adverse events will be followed up and trial discontinuation will be documented in the appropriate CRF pages. If possible, subjects who discontinue from the study before completion should undergo the assessments and procedures scheduled for the follow-up visits. Once discontinued participants may not resume trial treatment.

A patient may withdraw from the follow-up visits or they may withdraw their consent for any data collected to be used. Patients will be encouraged to allow data that have been collected before withdrawal to be used in the analyses. However, if consent to use data/ is also withdrawn, then these will be discarded. Patients withdrawing from the study will revert to their GP

The trial may be prematurely discontinued by the Sponsor, Chief Investigator, Regulatory Authority or Funder on the basis of new safety information or for other reasons given by the Data Monitoring Committee / Trial Steering Committee regulatory authority or ethics committee concerned.

The trial may also be prematurely discontinued due to lack of recruitment or upon advice from the Trial Steering Committee, who will advise on whether to continue or discontinue the trial and make a recommendation to the Sponsor. If the trial is prematurely discontinued, active participants will be informed and no further participant data will be collected.

9 Name and description of all drugs used in the trial

Over encapsulated sertraline 50mg and matching placebo

9.1 Treatment of subjects

The IMP will be over encapsulated and the placebo will be an identical capsule filled with an inert excipient. The placebo capsule will exactly match the encapsulated IMP in dimensions and appearance, such that allocation concealment and blinding of the trial is maintained. The IMP will be over encapsulated and the placebo manufactured by a UK MIA(IMP) licence holder.

Participants will be asked about adherence at all the follow-up points It will be requested that empty packaging and unused medicines are returned as described in section 10.8.

9.2 Concomitant medication

Sertraline should not be administered concomitantly with Monoamine Oxidase (MAO) inhibitors or within two weeks after discontinuation of MAO inhibitor therapy. Likewise at least two weeks should pass before patients treated with sertraline should be treated with MAO inhibitors. Participants in this study will not be treated with MAO inhibitors and GPs will be advised to wait at least 2 weeks after stopping the trial medication before starting an MAO inhibitor. Sertraline should not be administered concomitantly with pimozide.

Co-administration with other serotonergic active substances (Ltryptophan, triptans, tramadol, linezolid, lithium and St. John's Wort – Hypericum perforatum – preparations) may lead to an incidence of serotonin associated effects and participants will be advised not to take any of these medications for the duration of the trial.

Sertraline may increase the sedating properties of benzodiazepines and other sedatives (notably most antipsychotics, antihistamine H1 antagonists, opioids). The participants will be advised that caution should be exercised when these medicinal products are prescribed together with sertraline and they should be alert to the possibility of over sedation.

Sertraline may increase the CNS depressant effect of alcohol. Participants will therefore be advised to be cautious in their intake of alcohol while taking sertraline.

Co-administration of sertraline 200 mg daily with warfarin resulted in a small but statistically significant increase in prothrombin time, which may in some rare cases unbalance the INR value. If the participant is taking warfarin the general practitioner will be asked to monitor prothrombin time when sertraline therapy is initiated or stopped and provide the results to the local PI.

The risk of bleeding may be increased when medicines acting on platelet function (e.g. NSAIDs, acetylsalicylic acid and ticlopidine) or other medicines that might increase bleeding risk are concomitantly administered with SSRIs, including sertraline. The PI will decide whether the participant should be included in the study if they are also taking any NSAIDs or other medicines that might increase bleeding risk.

Participants will be allowed to take hypnotic medication along with the trial medication. The general practitioner will confirm other medication that the participant is taking in order to assess contraindications to sertraline before the baseline assessment. The participants will be asked about any concomitant medication at all follow up points.

10 Investigational Medicinal Product

10.1 Name of IMP

Sertraline 50mg

Placebo

10.2 Summary of findings from non-clinical studies

May be found in the Summary of Product Characteristics.

10.3 Summary of findings from clinical studies

Sertraline is an effective antidepressant for people with a depressive disorder. However, in cases of less severe depression there is still uncertainty about the severity and duration of symptoms that are associated with a clinically significant treatment response. These data have been reviewed above.

10.4 Summary of known and potential risks and benefits

The adverse effects of sertraline have been well described and included in the Patient Information Leaflet supplied by the manufacturer and in the British National Formulary, www.medicines.org.uk/emc.

Table : Adverse Reactions

**Very Common**<br><br>(≥1/10)	**Common**<br><br>(≥1/100 to <1/10)	**Uncommon**<br><br>(≥1/1000 to <1/100)	**Rare**<br><br>(≥1/10000 to <1/1000)	**Very. rare**<br><br>(<1/10000)	**Frequency not Known**
Infections and Infestations					
	Pharyngitis	Upper Respiratory Tract Infection, Rhinitis	Diverticulitis, Gastroenteritis, Otitis Media		
Neoplasms benign, malignant (including cysts and polyps)					
			Neoplasm†		
Blood and lymphatic system disorders					
			Lymphadenopathy		Leucopenia, Thrombocytopenia
Immune system disorders					
					Anaphylactoid Reaction, Allergic Reaction, Allergy
Endocrine disorders					
					Hyperprolactinaemia, Hypothyroidism and syndrome of inappropriate ADH secretion
Metabolism and Nutrition Disorders					
	Anorexia, Increased Appetite*		Hypercholesterolaemia, Hypoglycaemia		Hyponatremia<br><br>diabetes mellitus, hyperglycaemia

Psychiatric Disorders					
Insomnia (19%)	Depression*, Depersonalisation, Nightmare, Anxiety*, Agitation*, Nervousness, Libido Decreased*, Bruxism	Hallucination*, Euphoric Mood*, Apathy, Thinking Abnormal	Conversion Disorder, Drug Dependence, Psychotic disorder*, Aggression*, Paranoia, Suicidal Ideation/behaviour***, Sleep Walking, Premature Ejaculation		Paroniria
Nervous System Disorders					
Dizziness (11%), Somnolence (13%), Headache (21%)*	Paraesthesia*, Tremor, Hypertonia, Dysgeusia, Disturbance in Attention,	Convulsion*, Muscle Contractions Involuntary*, Coordination Abnormal, Hyperkinesia, Amnesia, Hypoaesthesia*, Speech Disorder, Dizziness Postural, Migraine*	Coma*, Choreoathetosis, Dyskinesia, Hyperaesthesia, Sensory Disturbance		Movement Disorders (including extrapyramidal symptoms such as hyperkinesia, hypertonia, dystonia, teeth grinding or gait abnormalities), Syncope.<br><br>Also reported were signs and symptoms associated with Serotonin Syndrome or Neuroleptic Malignant Syndrome: In some cases associated with concomitant use of serotonergic drugs that included agitation, confusion, diaphoresis, diarrhoea, fever, hypertension, rigidity and tachycardia.<br><br>Akathisia and psychomotor restlessness (see section 4.4), Cerebrovascular Spasm (including reversible cerebral vasconstriction syndrome and Call-Fleming syndrome).
Eye Disorders					

	Visual Disturbance		Glaucoma, Lacrimal Disorder, Scotoma, Diplopia, Photophobia, Hyphaema, Mydriasis*		Vision Abnormal, Pupils Unequal
Ear and Labyrinth Disorders					
	Tinnitus*	Ear Pain			
Cardiac Disorders					
	Palpitations*	Tachycardia	Myocardial Infarction, Bradycardia, Cardiac Disorder		
Vascular Disorders					
	Hot flush*	Hypertension*, Flushing	Peripheral Ischaemia		Abnormal Bleeding (such as epistaxis, gastrointestinal bleeding or haematuria)
Respiratory, Thoracic, and Mediastinal Disorders					
	Yawning*	Bronchospasm*, Dyspnoea, Epistaxis	Laryngospasm, Hyperventilation, Hypoventilation, Stridor, Dysphonia, Hiccups		Interstitial Lung Disease
Gastrointestinal Disorders					
Diarrhoea (18%), Nausea (24%), Dry Mouth (14%)	Abdominal Pain* Vomiting*, Constipation* Dyspepsia, Flatulence	Oesophagitis, Dysphagia, Haemorrhoids, Salivary Hypersecretion, Tongue Disorder, Eructation	Melaena, Haematochezia, Stomatitis, Tongue ulceration, Tooth Disorder, Glossitis, Mouth Ulceration		Pancreatitis
Hepatobiliary Disorders					

			Hepatic Function Abnormal		Serious liver events (including hepatitis, jaundice and liver failure)
Skin and Subcutaneous Tissue Disorders					
	Rash*, Hyperhidrosis	Periorbital Oedema*, Purpura*, Alopecia*, Cold Sweat, Dry skin, Urticaria*	Dermatitis, Dermatitis Bullous, Rash Follicular, Hair Texture Abnormal, Skin Odour Abnormal		Rare reports of severe cutaneous adverse reactions (SCAR): e.g. Stevens-Johnson syndrome and epidermal necrolysis, Angioedema, Face Oedema, Photosensitivity, Skin Reaction, Pruritus
Musculoskeletal and Connective Tissue Disorders					
	Myalgia	Osteoarthritis, Muscular Weakness, Back Pain, Muscle Twitching	Bone Disorder		Arthralgia, Muscle Cramps
Renal and Urinary Disorders					
		Nocturia, Urinary Retention*, Polyuria, Pollakiuria, Micturition disorder	Oliguria, Urinary Incontinence*, Urinary Hesitation		
*Reproductive System and Breast Disorders***					
Ejaculation Failure (14%)	Sexual Dysfunction, Erectile Dysfunction	Vaginal Haemorrhage, Female Sexual Dysfunction	Menorrhagia, Atrophic Vulvovaginitis, Balanoposthitis, Genital Discharge, Priapism*, Galactorrhoea*		Gynaecomastia, Menstrual Irregularities
General Disorders and Administration Site Conditions					

Fatigue (10%)*	Chest Pain*	Malaise*, Chills, Pyrexia*, Asthenia*,Thirst	Hernia, Drug Tolerance Decreased, Gait Disturbance		Oedema Peripheral
Investigations					
		Weight Decreased*, Weight Increased*	Alanine Aminotransferase Increased*, Aspartate Aminotransferase Increased*, Semen Abnormal		Abnormal Clinical Laboratory Results, Altered Platelet Function, Increased Serum Cholesterol
Injury and poisoning					
			Injury		
Surgical and medical procedures					
			Vasodilation Procedure		

If adverse experience occurred in depression, OCD, panic disorder, PTSD and social anxiety disorder, body term reclassified by depression studies body term.

† *One case of neoplasm was reported in one patient receiving sertraline compared with no cases in the placebo arm.*

** these adverse reactions also occurred in postmarketing experience*

*** the denominator uses the number of patients in that sex group-combined: sertraline (1118 males, 1424 females) placebo (926 males, 1219 females)*

For OCD, short term, 1-12 week studies only

**** Cases of suicidal ideation and suicidal behaviours have been reported during sertraline therapy or early after treatment discontinuation*

10.5 Description and justification of route of administration and dosage

Sertraline will be used according to the approved dose and method of administration.

10.6 Dosages, dosage modifications and method of administration

Sertraline 1 x 50mg oral capsule per day for 1 week followed by 2 x 50mg if tolerated. If the IMP is not tolerated the participant should reduce to the tolerated dose. With the permission of the PI it can be increased to 3 capsules, usually after 4 weeks.

Stopping the medication: following the 12 week follow up the participants will be asked to start tapering their medication. If on 2 capsules, they will be recommended to take 1 capsule for 1 week followed by 1 capsules on alternate days for the second week. If on 3 capsules they will be asked to reduce to 2 capsules for 5 days, 1 capsule for 5 days and then 1 capsule on alternate days for the final 4 days. They can consult their general practitioner after the 12 week assessment and request unblinding (section 8.3) and this will enable them to be advised about any further treatment.Preparation and labelling of Investigational Medicinal Product

The labelling of medication packs will be labelled in accordance with applicable Regulations and MHRA approved. Each Medication Pack will have a Medicine ID number, randomly generated to ensure sertraline and placebo medicine packs are indistinguishable (e.g. avoid all placebo packs being assigned an odd number) and thus maintain allocation concealment. This random number will be generated by the CTU and provided to the manufacturer who will use as a unique identifier for the IMP packages and to the randomisation/ code break service.

10.7 IMP accountability

The manufacturer will ship labelled and numbered packages to a pharmacy (to be decided) where the trial medication will be stored under controlled conditions. Storage will be secure, and there will be a delegation log for access, for which the pharmacy will take responsibility. The pharmacy will dispense individual patient packs and oversee the packaging and posting of those packs. After randomisation patient packs containing 6weeks supply (90 capsules) of the trial medication will be posted by recorded delivery to the participant's GP surgery, or, in exceptional circumstances, their homes. After the 6 week assessment has been completed a further 4 weeks medication and final 4 weeks medication including sufficient for the tapering period (90 capsules) will be posted by recorded delivery. All deliveries will be logged to ensure drug accountability. The trial medication will be shipped and stored in conditions in line with manufacturer's stability data.

Full IMP accountability records will be maintained in the trial, receipt, dispensing, distribution, return and destruction records will be maintained at the dispensing pharmacy. When the IMP arrives at the general practice it will be kept in a secure locked cabinet until collected by the participant. The receipt and collection of the IMP will be logged by the research team further details will be included in the IMP management plan for the trial.

Any used medicine that is returned will be passed to the pharmacy for accountability before destruction following authorisation by the Sponsor in line with the pharmacy medication disposal SOP.

10.10 Source of IMPs including placebo

Commercially available Sertraline 50mg will be over-encapsulated in identically appearing placebo capsules and will be manufactured on behalf of the Sponsor by the a UK MIA(IMP) holder. Capsules will be repackaged in bottles labelled as per annex 13 that will disguise the identity of medicine to maintain the blinding of the product.

10.11 Dose modifications

See 10.6.

10.12 Assessment of compliance

IMP adherence will be assessed by counting the tablets returned by the participant and by asking them questions about their adherence. These data will be recorded in the CRF. Non-compliance to the protocol study procedures will be documented by the investigator and reported to the Sponsor as agreed. The participants will be asked to complete all assessments even if they do not take the study medication. Information on adherence will be used in the final analysis plan that will be agreed by the Trial Steering Committee.

10.13 Post-trial IMP arrangements

Following completion of the study the participants will be asked to consult with their general practitioner to discuss any further treatment that might be necessary. Further trial medication will not be supplied.

11 Recording and reporting the adverse events and reactions

11.1 Definitions

Term	Definition
Adverse Event (AE)	Any untoward medical occurrence in a patient or clinical trial subject administered a medicinal product and which does not necessarily have a causal relationship with this treatment.
Adverse Reaction (AR)	Any untoward and unintended response in a subject to an investigational medicinal product which **is related** to any dose administered to that subject.<br>*This includes medication errors, uses outside of protocol (including misuse and abuse of product)*
Serious adverse event (SAE), serious adverse reaction (SAR) or unexpected serious adverse reaction	Any adverse event, adverse reaction or unexpected adverse reaction, respectively, that:<br>• results in death,<br>• is life-threatening,<br>• requires hospitalisation or prolongation of existing hospitalisation,<br>• results in persistent or significant disability or incapacity, or<br>• consists of a congenital anomaly or birth defect
Important Medical Event	These events may jeopardise the subject or may require an intervention to prevent one of the above characteristics/consequences. Such events should also be considered 'serious'.

Unexpected adverse reaction	An adverse reaction the nature and severity of which is not consistent with the information about the medicinal product in question set out:<br><br>(a) in the case of a product with a marketing authorization, in the summary of product characteristics for that product,<br>(b) in the case of any other investigational medicinal product, in the investigator's brochure relating to the trial in question.
SUSAR	Suspected Unexpected Serious Adverse Reaction

11.2 Recording adverse events

All AEs of special interest will be recorded by a structured assessment only included in the 2, 6 and 12 week follow up assessments. If a participant consults the general practitioner with a known adverse event it will be recorded in the medical notes only but not communicated to the PI unless specifically requested. This will include any adverse events that occur during the tapering period after the 12 week assessment.

As this trial is a phase IV trial of a licensed medication used within its licensed indication with a well-established safety profile, AEs will not be recorded in the CRF apart from those AEs of special interest included in the follow up assessments.

11.3 Assessments of Adverse Events

Each adverse event will be assessed for the following criteria:

11.3.1 Severity

Category	Definition
Mild	The adverse event does not interfere with the volunteer's daily routine, and does not require intervention; it causes slight discomfort
Moderate	The adverse event interferes with some aspects of the volunteer's routine, or requires intervention, but is not damaging to health; it causes moderate discomfort
Severe	The adverse event results in alteration, discomfort or disability which is clearly damaging to health

11.3.2 Causality

The assessment of relationship of adverse events to the administration of IMP is a clinical decision based on all available information at the time of the completion of the case report form. The following categories will be used to define the causality of the adverse event:

Category	Definition
Definitely:	There is clear evidence to suggest a causal relationship, and other possible contributing factors can be ruled out.
Probably:	There is evidence to suggest a causal relationship, and the influence of other factors is unlikely
Possibly	There is some evidence to suggest a causal relationship (e.g. the event occurred within a reasonable time after administration of the trial medication). However, the influence of other factors may have contributed to the event (e.g. the patient's clinical condition, other concomitant events).
Unlikely	There is little evidence to suggest there is a causal relationship (e.g. the event did not occur within a reasonable time after administration of the trial medication). There is another reasonable explanation for the event (e.g. the patient's clinical condition, other concomitant treatments).
Not related	There is no evidence of any causal relationship.
Not Assessable	Unable to assess on information available.

11.3.3 Expectedness

Category	Definition
Expected	An adverse event that is classed in nature as serious and which is consistent with the information about the IMP listed in the Investigator Brochure (or SmPC if Licensed IMP) **or clearly defined in this protocol.**
Unexpected	An adverse event that is classed in nature as serious and which is not consistent with the information about the IMP listed in the Investigator Brochure (or SmPC if Licensed IMP)

The reference document to be used to assess expectedness against the IMP is the SPC by Bristol Laboratories Ltd.

11.3.4 Seriousness

Seriousness as defined for an SAE in section 11.1.

Collection, recording and reporting of adverse events (including serious and non-serious events and reactions) to the sponsor will be completed according to the sponsor's SOP(INV/S05).

11.4 Procedures for recording and reporting Serious Adverse Events

Patients will be asked about SAEs at each visit using open ended questions.

All SAEs will be recorded in the CRF and the sponsor's AE log. The AE log will be reportable to the sponsor once a year.

The PI or delegate at trial sites will inform the CI by fax of any SAEs who will report onwards to the Sponsor as below.

All SAEs will be reported to the Sponsor on a SAE form. The CI or an appropriate delegated member of staff will complete the SAE form and fax to the sponsor on 020 3108 2312 or email on sae@ucl.ac.uk within 24 hours of his/her becoming aware of the event. The CI/PI will respond to any SAE queries raised by the Sponsor as soon as possible.

The CI/PI may contact the patient's GP, depending upon the nature of the SAE, to obtain more information regarding the adverse event. This information must be faxed over to site and placed in the diary card at end of trial.

All SUSARs must be notified to the sponsor within 24 hours according to the Sponsor's written SOP.

All SARs will be reportable to the sponsor until 30 days after last IMP administration.

11.4.1 Notification of deaths

All deaths, including deaths deemed unrelated to the IMP will be reported to the sponsor within 24 hours of the CI or PI being made aware of the event.

11.4.2 Reporting SUSARs

The sponsor will notify the main REC and MHRA of all SUSARs. SUSARs that are fatal or life-threatening must be notified to the MHRA and REC within 7 days after the sponsor has learned of them. Other SUSARs must be reported to the REC and MHRA within 15 days after the sponsor has learned of them.

Please refer to section 8.3 for unblinding in the case of a SUSAR

11.4.3 Multisite responsibilities

The PIs will inform the CI by fax of any SAEs so that the CI can report any SAEs to the sponsor within the required time interval. All trial sites will be responsible for reporting other safety information in CRFs and logs to facilitate DSUR reporting.

Any SUSARs reported in the trial will be circulated to all trial sites.

11.4.4 Development Safety Update Reports

The sponsor will provide the main REC and the MHRA with Development Safety Update Reports (DSUR) which will be written in conjunction with the trial team and the Sponsor's office. The report will be submitted within 60 days of the Developmental International Birth Date (DIBD) of the trial each year until the trial is declared ended. This will be done in accordance with the sponsor's SOP.

11.4.5 Annual progress reports

An annual progress report (APR) will be submitted to the REC within 30 days of the anniversary date on which the favourable opinion was given, and annually until the trial is declared ended.

The chief investigator will prepare the APR.

11.4.6 Pregnancy

Should a study participant become pregnant whilst in the Trial, the pregnancy, the patient will be followed up until term or termination. The BNF advice is that the benefits of the IMP should outweigh the risks if it were to be used in pregnancy. At baseline, we will exclude potential patients who have a positive pregnancy test result or are intending to become pregnant.

11.4.6 Overdose

Due to the nature of the patient's condition, there is a possibility of overdoses occurring.

On the evidence available, sertraline has a wide margin of safety in overdose. Overdoses of sertraline alone of up to 13.5 g have been reported. Deaths have been reported involving overdoses of sertraline, primarily in combination with other drugs and/or alcohol. Therefore, any over dosage should be medically treated aggressively.

Symptoms

Symptoms of overdose include serotonin-mediated side effects such as somnolence, gastrointestinal disturbances (such as nausea and vomiting), tachycardia, tremor, agitation and dizziness. Less frequently reported was coma.

Treatment

There are no specific antidotes to sertraline. Establish and maintain an airway and ensure adequate oxygenation and ventilation, if necessary. Activated charcoal, which may be used with a cathartic, may be as or more effective than lavage, and should be considered in treating overdose. Induction of emesis is not recommended. Cardiac and other vital sign monitoring is recommended, along with general symptomatic and supportive measures. Due to the large volume of distribution of sertraline, forced diuresis, dialysis, haemoperfusion and exchange transfusion are unlikely to be of benefit.

Any overdoses will be reported to the sponsor within 24 hours of being made of the event and recorded on the deviation log and the CRF

Overdoses can be observed from pill counts, diary card and the patient's comments.

If an SAE is associated with the overdose the CI/PI will complete the SAE report form detailing the overdose information.

11.4.7 Reporting Urgent Safety Measures

If any urgent safety measures are taken the PI/Sponsor shall immediately and in any event no later than 3 days from the date the measures are taken, give written notice to the MHRA and the relevant REC of the measures taken and the circumstances giving rise to those measures.

11.5 The type and duration of the follow-up of subjects after adverse events.

In the event that a subject suffers from a SAE, we will advise the subject to contact their GP immediately and follow up with the subject until a resolution or stabilisation is reached.

Events and reactions will be regarded as not related to the IMP if they occur more than 2 weeks after stopping the IMP.

Any SUSAR related to the IMP will be reported to the Sponsor irrespective of how long after IMP administration the reaction has occurred.

11.5.1 Notification of Serious Breaches to GCP and/or the protocol (SPON/S15)

A “serious breach” is a breach which is likely to effect to a significant degree:

(a) the safety or physical or mental integrity of the subjects of the trial; or

(b) the scientific value of the trial.

The sponsor of a clinical trial shall notify the licensing authority in writing of any serious breach of –

(a) the conditions and principles of GCP in connection with that trial; or (b) the protocol relating to that trial, as amended from time to time, within 7 days of becoming aware of that breach.

The sponsor will be notified immediately of any case where the above definition applies during the trial conduct phase. The sponsor’s SOP on the ‘Notification of violations, urgent safety measures and serious breaches’ will be followed.

12 Data management and quality assurance

12.1 Confidentiality

All data will be handled in accordance with the UK Data Protection Act 1998.

The Case Report Forms (CRFs) will not bear the subject’s name or other personal identifiable data. The subject’s initials, date of birth and trial identification number, will be used for identification.’

12.2 Data collection tools and source document identification

Participants will be explained before consent the requirement to attend all follow up assessments. The 2, 6 and 12 week assessments will be face to face appointments. We will write letters, email and telephone the participants in order to maximise the response rate.

CISR – Revised clinical interview schedule. This is a self-administered assessment of psychiatric symptoms including depression. It generates a computer file that will then be incorporated into the main CRF held on a database.

PHQ9, BDI-II, SF12, EQ5D, GAD7 – Are self-administered questionnaire will either be administered in a paper and pencil format or in computerised format. The electronic or paper records will then be transferred to the main CRF held on a database.

Emotional processing tasks.

Emotional memory task: the participant will be asked to write down the words that are remembered. These will be coded as correct or incorrect by the delegated member of staff and that information will be transferred onto the database.

Emotion recognition task: the participant carries out the task on a PC and this generates an electronic file that contains responses and reaction times. The file will be stored and achieve electronically. A selection of this information, word recall scores, will then be transferred to the database.

It will be the responsibility of the investigator to ensure the accuracy of all data entered in the CRFs. The delegation log will identify all those personnel with responsibilities for data collection and handling, including those who have access to the trial database. Source data will be maintained at each trial site for each patient and will be defined prior to the start of data collection at each site.

All data at site will be handled according to the Data Protection Act 1998. Any patient identifiers will be removed prior to the submission of documents to the Sponsor or coordinating centre.

12.3 Data handling and analysis

A database will be prepared by CTU and Sealed Envelope that will include facility for data entry (eCRF). This will be accessed via a secure website to allow data to be entered from all sites.

We will adopt a data management SOP as approved by the sponsor. The local PI will be responsible for the data quality.

All electronic data will be handled according to the Data Protection Act 1998 as well as UCL Information Security Policy and Trust Information Governance Policy.

Data analysis will be performed under the supervision of the trial statistician. Data analysis will be completed independently from data entry. A data analysis plan will be agreed by the Trial Steering Committee before the database is locked.

13 Record keeping and archiving

Archiving will be authorised by the Sponsor following submission of the end of study report. Archiving will be carried out in line with Sponsor SOP.

The Chief Investigator will be responsible for the secure archiving of essential trial documents (for each site, if multi-site trial) and the trial database as per local trust policy. All essential documents will be archived for a minimum of 5 years after completion of trial.

Destruction of essential documents will require authorisation from the Sponsor.

14 Statistical Considerations

Prof Tim Peters, University of Bristol, is the trial statistician who will be responsible for all statistical aspects of the trial from design through to analysis and dissemination.

14.1 Outcomes

14.1.1 Primary outcome

Depressive symptoms measured using the PHQ9 at 6 weeks as a continuous outcome.

14.1.2 Secondary outcomes

Depressive symptoms with the PHQ9 at 2 & 12 weeks as a continuous outcome and 2, 6 & 12 weeks as a binary outcome. BDI-II as an alternative measure of depressive symptoms as continuous and binary at all follow up points. Anxiety symptoms measured using the GAD7 as a continuous and binary measure at all follow-up points. Quality of life assessed using the EQ5D and SF12. Economic costs associated with the treatment. Emotional processing tasks at and 6 weeks.

14.2 Sample size and recruitment

14.2.1 Sample size calculation

The primary analysis will be an interaction term between severity of symptoms an duration of symptoms and treatment allocation.

One approach towards deciding on a clinically important difference is to use the NICE guidelines suggestion that 3 points on the HAMD scale is clinically important.(37) The standard deviations in depression trials for the HAMD have a median value of about 8.5.(1) Three points on the HAMD will then correspond to 0.35 standard deviations. If we assume this difference, we would need 129 analysed in each group for 80% power at the 5% level to detect this difference or 172 in each group for 90% power.

We will use the results from Brookes et al to estimate the power for the interaction test. For a situation where the interaction term size is 1.3 times the main effect, there is an inflation factor of 2 (i.e. doubling the sample size for the main effect) to retain the same power and significance as for the main effect. In this circumstance, the interaction would therefore vary between 0.27 and 0.46 standard deviations and the required sample size would be 516 for 80% power 5% significance. Assuming 90% follow-up (91% in GENPOD at 6 weeks)(10) we would need to recruit 573 participants for 80% power, 653 for 85% and 764 for 90% power (all at 5% significance).

Clearly there are a number of unknowns in these calculations concerning the interaction term. For example, if the clinically important difference were 0.3 standard deviations, the ensuing sample size would be 777 for 80% power and 5% significance. For a clinically important difference of 0.4 standard deviations, the sample size for 90% power and 5% significance would be 582. In the light of these considerations we have set our target as 653.

14.2.2 Planned recruitment rate

We propose to initially recruit 30 participants in Bristol as the pilot phase. This will provide an estimate of the likely referral rate and follow up rates.

It is recognised that this study may be a challenging trial for recruitment. THREAD had difficulty in recruitment with a final size of 220. We hope to address this concern in the following ways. This trial will have broader criteria for inclusion as THREAD excluded people who scored high or low on the HAMD or who had been on antidepressants at any time in the previous 12 months (we are suggesting 8 weeks). These criteria led to the exclusion of 137 (23%) of those referred to THREAD. In THREAD 77 (13%) of the referrals to the study refused randomisation because they did not want antidepressants. These people will be identified early on by telephone screening to improve the efficiency of recruitment. It is believed that the reduced time of the trial might make antidepressants more acceptable to participants. Finally a qualitative study of recruitment as described below will also be conducted to inform if any changes or adjustments are required in this protocol.

Sample size calculation suggests a sample of 653 participants is needed. Approximately 200 practices would be required for 18 months and on average for each practice to refer 0.35 per month of whom 52% randomised. This referral rate is that achieved in the GENPOD study which recruited 601 people to a randomised study of two antidepressants. Also, given the broad entry criteria it is expected that almost everyone referred to the study will be randomised so the 52% recruitment after baseline is pessimistic. 24 months for recruitment is being allowed partly to enable time to recruit all the practices. All centres have existing Primary Care Research Networks and experience of recruiting to such trials.

14.3 Statistical analysis plan

14.3.1 Summary of baseline data and flow of patients

We will follow the CONSORT guidelines in reporting and analysing our data. We will create a flow chart that will provide the number of potential participants that were screened, eligible, randomised and followed up at each time point.

14.3.2 Primary outcome analysis

Our primary outcome will be the PHQ9 at 6 weeks. The primary analysis will be studying interaction terms within a linear regression model between the randomised treatment and baseline severity of symptoms and baseline duration of symptoms. The severity of symptoms will be measured using the sum of the fatigue, concentration, sleep, depression and depressive thoughts section of the CISR.   The CISR also asks about the duration of depressive symptoms. We will use a linear regression model with adjustment for baseline scores on the PHQ9 and the minimisation variables. We will adopt an intention to treat approach. We will carry out analyses to investigate the likely impact of any missing data using multiple imputation.

We will also carry out exploratory analyses to determine the most likely combination of severity of symptoms and duration that is associated with a clinically significant improvement.

14.3.3 Secondary outcome analysis

A secondary analysis will be to examine whether there is a difference between antidepressant and placebo irrespective of baseline severity or duration. We are expecting that clustering by practice will be negligible given the pharmacological nature of the intervention. We are currently carrying out work within the overall PANDA programme to determine the minimum clinically important difference and will use these results to inform this analysis.

We will also carry out the economic analysis. NHS and Personal Social Services resource use will be valued using local and, where available, national unit cost data. The cost-effectiveness analysis will evaluate the efficiency of sertraline stratified by baseline severity and duration of symptoms. We will calculate net monetary benefit (NMB)(36) of treatment for individual patients, based on standard NICE willingness to pay thresholds. The advantage of using the NMB approach rather than a more conventional incremental cost effectiveness ratio is that we can use net benefit regression to explore the relationship between symptom severity and duration and economically important differences in patient costs and outcomes in an analogous fashion to the statistical analysis described above.

14.3.4 Sensitivity and other planned analyses

The main sensitivity analysis will investigate the likely impact of missing data using multiple imputation methods.

14.4 Randomisation methods

Randomisation will be using a computer generated code on a web based platform. The randomisation ratio will be 1:1 and will be stratified by centre and severity and duration of depressive symptoms. There will be random permutation of the block size. People who withdraw will not be replaced as the target sample size has allowed for attrition.

14.5 Interim analysis

There will be no interim analyses apart from those requested by the DSMC.

14.6 Other statistical considerations

A more detailed analysis plan will be agreed with the Trial Steering committee. We will publish a protocol giving an outline of the analysis plan before the study is completed.

15 Name of Committees involved in trial

A trial management group will meet regularly at minuted meetings to review AE logs, recruitment rates and all other aspects of the trial.

There will be a Trial Steering Committee (TSC) whose external members will include a clinician, a statistician and a PPI representative. The TSC will advise on the composition of a Data Safety and Monitoring Committee (DSMC).

Terms of reference will be in place for each committee.

16 Direct Access to Source Data/Documents

The investigator(s)/ institution(s) will permit trial-related monitoring, audits, REC review, and regulatory inspection(s), providing direct access to source data/documents. Trial participants are informed of this during the informed consent discussion. Participants will consent to provide access to their medical notes.

17 Ethics and regulatory requirements

The participants will be asked to give informed consent at baseline interview appointment.

The sponsor will ensure that the trial protocol, patient information sheet, consent form, GP letter and submitted supporting documents have been approved by the appropriate regulatory body (MHRA in UK) and a main research ethics committee, prior to any patient recruitment. The protocol and all agreed substantial protocol amendments, will be documented and submitted for ethical and regulatory approval prior to implementation.

Before the site can enrol patients into the trial, the Chief Investigator/Principal Investigator or designee will apply for NHS permission from their Trust Research & Development (R&D) and be granted written permission. It is the responsibility of the Chief Investigator/ Principal Investigator or designee at each site to ensure that all subsequent amendments gain the necessary approval. This does not affect the individual clinician’s responsibility to take immediate action if thought necessary to protect the health and interest of individual patients (see section 0 for reporting urgent safety measures).

Within 90 days after the end of the trial, the CI/Sponsor will ensure that the main REC and the MHRA are notified that the trial has finished. If the trial is terminated prematurely, those reports will be made within 15 days after the end of the trial.

The CI will supply the Sponsor with a summary report of the clinical trial, which will then be submitted to the MHRA and main REC within 1 year after the end of the trial.

18 Monitoring requirement for the trial

A Trial specific monitoring plan will be established for the study. The trial will be monitored with the agreed plan.

19 Finance

The study is funded by the NIHR Programme Grants for Applied Research RP-PG-0610-10048. The main trial funding is conditional on a submission to the funders in June 2014.

20 Insurance

University College London holds insurance against claims from participants for injury caused by their participation in the clinical trial. Participants may be able to claim compensation if they can prove that UCL has been negligent. However, as this clinical trial is being carried out in a hospital, the hospital continues to have a duty of care to the participant of the clinical trial. University College London does not accept liability for any breach in the hospital's duty of care, or any negligence on the part of hospital employees. This applies whether the hospital is an NHS Trust or otherwise.

Participants may also be able to claim compensation for injury caused by participation in this clinical trial without the need to prove negligence on the part of University College London or another party. Participants who sustain injury and wish to make a claim for compensation should do so in writing in the first instance to the Chief Investigator, who will pass the claim to the Sponsor's Insurers, via the Sponsor's office.

21 Publication policy

All proposed publications will be discussed with Sponsor prior to publishing other than those presented at scientific forums/meetings. Please refer to UCL publication policy.

22 Statement of compliance

The trial will be conducted in compliance with the approved protocol, the UK Regulations, EU GCP and the applicable regulatory requirement(s).

23 References

1. Jenkins R, Lewis G, Bebbington P, Brugha T, Farrell M, Gill B, et al. The National Psychiatric Morbidity Surveys of Great Britain: Initial findings from the Household Survey. Psychological Medicine. 1997;27:775-90.
2. Excellence NIfHaC. Depression (updated edition). London: The British Psychological Society and The Royal College of Psychiatrists, 2009.
3. Hyde J, Calnan M, Prior L, Lewis G, Kessler D, Sharp D. Reluctant medicalisers? A qualitative study exploring how general practitioners decide to prescribe antidepressants. British Journal of General Practice. 2005;55:755-62.
4. Depression: management of depression in primary and secondary care. Clinical Guideline 23. London: National Institute for Clinical Excellence; 2004. 1-63 p.
5. Cipriani A, Furukawa TA, Salanti G, Geddes JR, Higgins JP, Churchill R, et al. Comparative efficacy and acceptability of 12 new-generation antidepressants: a multiple-treatments meta-analysis. Lancet. 2009;373(9665):746-58.
6. Sinclair LI, Christmas DM, Hood SD, Potokar JP, Robertson A, Isaac A, et al. Antidepressant-induced jitteriness/anxiety syndrome: systematic review. Br J Psychiatry. 2009;194(6):483-90.
7. Hawton K, Bergen H, Simkin S, Cooper J, Waters K, Gunnell D, et al. Toxicity of antidepressants: rates of suicide relative to prescribing and non-fatal overdose. Br J Psychiatry. 2010;196(5):354-8.
8. Kirsch I, Deacon BJ, Huedo-Medina TB, Scoboria A, Moore TJ, Johnson BT. Initial severity and antidepressant benefits: a meta-analysis of data submitted to the Food and Drug Administration. PLoS Med. 2008;5(2):e45.
9. Khan A, Leventhal RM, Khan SR, Brown WA. Severity of depression and response to antidepressants and placebo: an analysis of the Food and Drug Administration database. JClinPsychopharmacol. 2002;22(1):40-5.
10. Fournier JC, DeRubeis RJ, Hollon SD, Dimidjian S, Amsterdam JD, Shelton RC, et al. Antidepressant drug effects and depression severity: a patient-level meta-analysis. JAMA. 2010;303(1):47-53.
11. Gibbons RD, Hur K, Brown CH, Davis JM, Mann JJ. Benefits from antidepressants: synthesis of 6-week patient-level outcomes from double-blind placebo-controlled randomized trials of fluoxetine and venlafaxine. Arch Gen Psychiatry. 2012;69(6):572-9.
12. Barbui C, Cipriani A, Patel V, yuso-Mateos JL, van OM. Efficacy of antidepressants and benzodiazepines in minor depression: systematic review and meta-analysis. Br J Psychiatry. 2011;198:11-6, sup.
13. Silva de LM, Hotopf M. A comparison of active drugs for the treatment of dysthymia. Cochrane Database Syst Rev. 2003(3):CD004047.
14. Anderson IM, Nutt DJ, Deakin JF. Evidence-based guidelines for treating depressive disorders with antidepressants: a revision of the 1993 British Association for Psychopharmacology guidelines.British Association for Psychopharmacology. J Psychopharmacol. 2000;14(1):3-20.
15. Pampallona S, Bollini P, Tibaldi G, Kupelnick B, Munizza C. Combined pharmacotherapy and psychological treatment for depression: a systematic review. Arch Gen Psychiatry. 2004;61(7):714-9.
16. Taylor MJ, Freemantle N, Geddes JR, Bhagwagar Z. Early Onset of Selective Serotonin Reuptake Inhibitor Antidepressant Action: Systematic Review and Meta-analysis. Arch Gen Psychiatry. 2006;63(11):1217-23.
17. Lewis G, Pelosi AJ, Araya R, Dunn G. Measuring psychiatric disorder in the community: a standardised assessment for use by lay interviewers. Psychological Medicine. 1992;22:465-86.
18. Lewis G. Assessing psychiatric disorder with a human interviewer or a computer. Journal of Epidemiology and Community Health. 1994;48:207-10.
19. Gilbody S, Richards D, Brealey S, Hewitt C. Screening for depression in medical settings with the Patient Health Questionnaire (PHQ): a diagnostic meta-analysis. J Gen Intern Med. 2007;22(11):1596-602.
20. Beck AT, Ward CH, Mendelsohn M, Mock J, Erbaugh J. An inventory for measuring depression. Archives of General Psychiatry. 1961;4:561-71.
21. Brazier J. Is the EQ-5D fit for purpose in mental health? Br J Psychiatry. 2010;197(5):348-9.
22. Spitzer RL, Kroenke K, Williams JB, Lowe B. A brief measure for assessing generalized anxiety disorder: the GAD-7. Arch Intern Med. 2006;166(10):1092-7.
23. Harmer CJ, Shelley NC, Cowen PJ, Goodwin GM. Increased positive versus negative affective perception and memory in healthy volunteers following selective serotonin and norepinephrine reuptake inhibition. AmJPsychiatry. 2004;161(7):1256-63.

24. Wiles N, Thomas L, Abel A, Ridgway N, Turner N, Campbell J, et al. Cognitive behavioural therapy as an adjunct to pharmacotherapy for primary care based patients with treatment resistant depression: results of the CoBalT randomised controlled trial. Lancet. 2013;381:375-84.
25. Lewis G, Mulligan J, Wiles N, Cowen P, Craddock N, Ikeda M, et al. Polymorphism of the 5-HT transporter and response to antidepressants: randomised controlled trial. Br J Psychiatry. 2011;198:464-71.

CONSORT 2010 checklist of information to include when reporting a randomised trial*

Section/Topic	Item No	Checklist item	Reported on page No
**Title and abstract**			
	1a	Identification as a randomised trial in the title	1
	1b	Structured summary of trial design, methods, results, and conclusions (for specific guidance see CONSORT for abstracts)	1
**Introduction**			
Background and objectives	2a	Scientific background and explanation of rationale	2
	2b	Specific objectives or hypotheses	1-2
**Methods**			
Trial design	3a	Description of trial design (such as parallel, factorial) including allocation ratio	3
	3b	Important changes to methods after trial commencement (such as eligibility criteria), with reasons	3
Participants	4a	Eligibility criteria for participants	3
	4b	Settings and locations where the data were collected	3
Interventions	5	The interventions for each group with sufficient details to allow replication, including how and when they were actually administered	3
Outcomes	6a	Completely defined pre-specified primary and secondary outcome measures, including how and when they were assessed	3-4
	6b	Any changes to trial outcomes after the trial commenced, with reasons	Appendix
Sample size	7a	How sample size was determined	4
	7b	When applicable, explanation of any interim analyses and stopping guidelines	
Randomisation:			
Sequence generation	8a	Method used to generate the random allocation sequence	3
	8b	Type of randomisation; details of any restriction (such as blocking and block size)	3
Allocation concealment mechanism	9	Mechanism used to implement the random allocation sequence (such as sequentially numbered containers), describing any steps taken to conceal the sequence until interventions were assigned	3
Implementation	10	Who generated the random allocation sequence, who enrolled participants, and who assigned participants to interventions	
Blinding	11a	If done, who was blinded after assignment to interventions (for example, participants, care providers, those	3

Section/Topic	Item No	Checklist item	Reported on page No
			3
		assessing outcomes) and how	3
	11b	If relevant, description of the similarity of interventions	
Statistical methods	12a	Statistical methods used to compare groups for primary and secondary outcomes	4
	12b	Methods for additional analyses, such as subgroup analyses and adjusted analyses	4 and Appendix
**Results**			
Participant flow (a diagram is strongly recommended)	13a	For each group, the numbers of participants who were randomly assigned, received intended treatment, and were analysed for the primary outcome	5
	13b	For each group, losses and exclusions after randomisation, together with reasons	5
Recruitment	14a	Dates defining the periods of recruitment and follow-up	6
	14b	Why the trial ended or was stopped	6
Baseline data	15	A table showing baseline demographic and clinical characteristics for each group	6-7
Numbers analysed	16	For each group, number of participants (denominator) included in each analysis and whether the analysis was by original assigned groups	6-7
Outcomes and estimation	17a	For each primary and secondary outcome, results for each group, and the estimated effect size and its precision (such as 95% confidence interval)	7-8
	17b	For binary outcomes, presentation of both absolute and relative effect sizes is recommended	7-8
Ancillary analyses	18	Results of any other analyses performed, including subgroup analyses and adjusted analyses, distinguishing pre-specified from exploratory	Appendix
Harms	19	All important harms or unintended effects in each group (for specific guidance see CONSORT for harms)	8
**Discussion**			
Limitations	20	Trial limitations, addressing sources of potential bias, imprecision, and, if relevant, multiplicity of analyses	9-10
Generalisability	21	Generalisability (external validity, applicability) of the trial findings	9-10
Interpretation	22	Interpretation consistent with results, balancing benefits and harms, and considering other relevant evidence	9-11
**Other information**			
Registration	23	Registration number and name of trial registry	1
Protocol	24	Where the full trial protocol can be accessed, if available	Appendix
Funding	25	Sources of funding and other support (such as supply of drugs), role of funders	1

*We strongly recommend reading this statement in conjunction with the CONSORT 2010 Explanation and Elaboration for important clarifications on all the items. If relevant, we also recommend reading CONSORT extensions for cluster randomised trials, non-inferiority and equivalence trials, non-pharmacological treatments, herbal interventions, and pragmatic trials. Additional extensions are forthcoming: for those and for up to date references relevant to this checklist, see www.consort-statement.org.
